# Identification of the novel activity-driven interaction between synaptotagmin 1 and presenilin 1 links calcium, synapse, and amyloid beta

**DOI:** 10.1186/s12915-016-0248-3

**Published:** 2016-03-31

**Authors:** Akira Kuzuya, Katarzyna M. Zoltowska, Kathryn L. Post, Muriel Arimon, Xuejing Li, Sarah Svirsky, Masato Maesako, Alona Muzikansky, Vivek Gautam, Dora Kovacs, Bradley T. Hyman, Oksana Berezovska

**Affiliations:** MassGeneral Institute for Neurodegenerative Disease, Department of Neurology, Massachusetts General Hospital, Harvard Medical School, Charlestown, MA 02129 USA; MGH Biostatistics Center, Massachusetts General Hospital, Boston, MA 02114 USA

**Keywords:** Synaptotagmin 1, Presenilin 1, Alzheimer’s disease, Beta amyloid

## Abstract

**Background:**

Synaptic loss strongly correlates with memory deterioration. Local accumulation of amyloid β (Aβ) peptide, and neurotoxic Aβ42 in particular, due to abnormal neuronal activity may underlie synaptic dysfunction, neurodegeneration, and memory impairments. To gain an insight into molecular events underlying neuronal activity-regulated Aβ production at the synapse, we explored functional outcomes of the newly discovered calcium-dependent interaction between Alzheimer’s disease-associated presenilin 1 (PS1)/γ-secretase and synaptic vesicle proteins.

**Results:**

Mass spectrometry screen of mouse brain lysates identified synaptotagmin 1 (Syt1) as a novel synapse-specific PS1-binding partner that shows Ca^2+^-dependent PS1 binding profiles in vitro and in vivo*.* We found that Aβ level, and more critically, conformation of the PS1 and the Aβ_42/40_ ratio, are affected by Syt1 overexpression or knockdown, indicating that Syt1 and its interaction with PS1 might regulate Aβ production at the synapse. Moreover, β-secretase 1 (BACE1) stability, β- and γ-secretase activity, as well as intracellular compartmentalization of PS1 and BACE1, but not of amyloid precursor protein (APP), nicastrin (Nct), presenilin enhancer 2 (Pen-2), or synaptophysin (Syp) were altered in the absence of Syt1, suggesting a selective effect of Syt1 on PS1 and BACE1 trafficking.

**Conclusions:**

Our findings identify Syt1 as a novel Ca^2+^-sensitive PS1 modulator that could regulate synaptic Aβ, opening avenues for novel and selective synapse targeting therapeutic strategies.

**Electronic supplementary material:**

The online version of this article (doi:10.1186/s12915-016-0248-3) contains supplementary material, which is available to authorized users.

## Background

Presenilin 1 (PS1) constitutes the catalytic site of the γ-secretase complex [1, 2] involved, together with β-secretase 1 (BACE1) [3], in amyloid precursor protein (APP) processing and amyloid β (Aβ) production. Abnormal Aβ production is implicated in the pathogenesis of Alzheimer’s disease (AD) — a neurodegenerative disorder characterized by progressive memory deterioration (reviewed in [4, 5]). Although the exact sequence of events occurring in the brain and causing AD is not well understood, it appears that Aβ accumulation occurs before the onset of cognitive decline [5, 6] and that synaptic loss most closely correlates with the memory impairments [7–9]. Moreover, traceable Aβ deposition could already be detected in the default mode network brain regions in cognitively normal adults [10], and experimentally induced synaptic activity was shown to increase Aβ production [11–13], indicating a link between synaptic activity and Aβ. Interestingly, the level of Aβ in the brain interstitial fluid is closely linked to synaptic vesicle (SV) exocytosis [14]. These findings suggest that Aβ can be produced locally at the synapse in an activity-dependent manner. However, molecular events and proteins involved in neuronal activity-modulated levels of synaptic Aβ remain largely unknown.

Synaptotagmin 1 (Syt1) is a key molecule regulating synaptic vesicle (SV) exocytosis in a calcium [Ca^2+^]-dependent manner [15, 16]. Syt1 contains two Ca^2+^-binding domains, C2A and C2B, and is one of the major calcium sensors at the synapse promoting SV exocytosis and neurotransmitter release in response to Ca^2+^ influx [16–18]. Interestingly, increased intracellular calcium levels also enhance production of Aβ and Aβ42 in particular [19–21], implying that Ca^2+^ influx may modulate the cleavage of APP by β- and γ-secretases. Still, little is known about the local synaptic effects of neuronal activity and Ca^2+^ on APP processing.

In the present study, we focused on, and searched for, potential neuronal activity-dependent modulators that can affect γ-secretase activity. We found that PS1 adopts a pathogenic “closed” conformation within minutes of Ca^2+^ influx triggered by glutamate or KCl treatment, suggesting a Ca^2+^-dependent mechanism. An unbiased proteomics screen of the mouse brain lysates in the presence or absence of Ca^2+^ identified Syt1 as a novel Ca^2+^-dependent PS1- interacting protein, showing robust Syt1-PS1 binding in the presence of high Ca^2+^. Furthermore, we found that Syt1 may regulate Aβ production via different mechanisms. We report that Syt1 modulates the architecture and activity of the PS1/γ-secretase complex affecting the Aβ species produced, is important for BACE1 maturation and stability, and is involved in trafficking and compartmentalization of BACE1 and PS1. Taken together, these data suggest that Syt1 acts as a novel pre-synaptic calcium-dependent interactor of the PS1/γ-secretase that can regulate APP processing and, thus, Aβ production/secretion at the synapse in an activity-dependent manner.

## Results

### PS1 conformation is dynamically regulated by Ca^2+^ influx in intact neurons

**Fig. 1:**
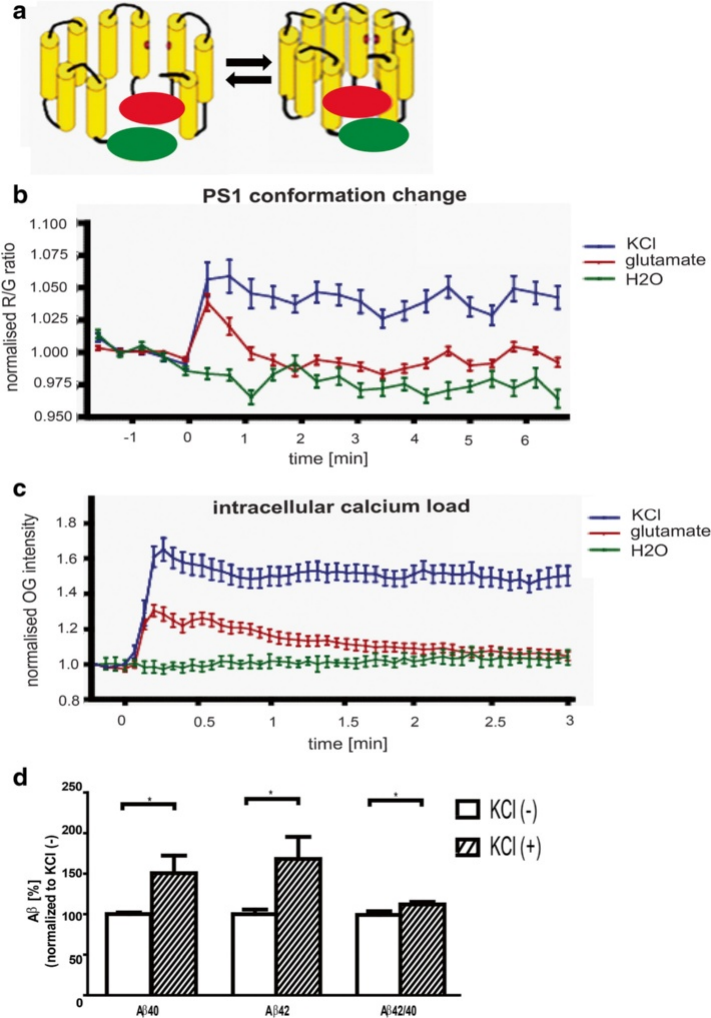
PS1 conformation and Aβ production change upon KCl or glutamate treatment. **a** Schematic representation of the “open” (left) and “closed” (right) PS1 conformation; *green* and *red circles* represent green fluorescent protein (GFP) and red fluorescence protein (RFP) fused to PS1 NT and L6-7, respectively, to generate FRET reporter probe. **b** Time-lapse recording of PS1 conformational changes in live neurons transfected with GFP-PS1-RFP FRET reporter probe and treated with KCl, glutamate, or water control. The Spectral FRET data are presented as a change in the RFP/GFP ratio, 50 to 100 neurons were analyzed for each condition; the graph shows mean ± SEM; detailed statistical analysis of the Spectral FRET data evaluation is described in the Methods. **c** Time-lapse recording of Oregon Green 488 BAPTA-1 AM fluorescence intensity changes reflecting intracellular calcium load in primary neurons treated with KCl, glutamate, or water control. Three independent experiments, mean ± SEM. **d** ELISA measurements of secreted Aβ40 and Aβ42 in conditioned-medium collected from KCl (KCl+) or H_2_O (KCl-) treated mouse cortical neurons. The Aβ levels determined in pmol were normalized to the amount of total protein [g] extracted from the cells in the corresponding well. Data are presented as mean% ± SEM, *n* = 4; 100 % = 91.11 pmol/g for Aβ40 and 11.04 pmol/g for Aβ42. Statistical significance was determined using the Mann-Whitney *U* test, *p < 0.05. *PS1* presenilin 1, Aβ amyloid β, *NT* N-terminus, *FRET* Förster Resonance Energy Transfer

Neuronal activation is reported to modulate Aβ production and elevate the Aβ42/40 ratio [13, 19, 21]. The latter correlates with distinct conformation of the PS1/γ-secretase [22–25]. To probe for possible dynamic changes in PS1/γ-secretase in live cells in response to neuronal stimulation we used our previously developed ratiometric spectral Förster Resonance Energy Transfer (FRET) assay that utilizes GFP-PS1-RFP (G-PS1-R) as a reporter of PS1 conformation (Fig. 1a). As determined previously, the G-PS1-R protein can traffic through the secretory pathway to the plasma membrane, shows similar subcellular distribution to that of endogenous PS1, can be incorporated into the γ-secretase complex, is efficiently endoproteolyzed, and reconstitutes the γ-secretase enzymatic activity in PS1/2 double knockout mouse embryonic fibroblasts (MEF) [25]. The change in the proximity between RFP and GFP fluorophore-tagged PS1 loop- and NT-domains (FRET efficiency) corresponds to the change in the ratio of RFP (598 nm) to GFP (513 nm) fluorescence intensity (R/G ratio). The higher the R/G ratio the closer the two domains are, indicating so-called “closed” PS1 conformation.

Primary neurons (12–14 days in vitro [DIV]) were transfected with the G-PS1-R and imaged every ~30 seconds prior to and after KCl bath treatment inducing membrane depolarization. We detected a rapid increase in the R/G ratio within the first minute of the stimulation, which lasted for at least 30 minutes (Fig. 1b and Additional file 1). This suggests that PS1 has a dynamic structure that responds rapidly to KCl treatment by changing the NT-loop proximity. To confirm this finding, we used another stimulus, glutamate (Glu) that was applied transiently by puffing it directly onto the imaged neuron. Again, a rapid increase in the R/G ratio was observed, indicating a change in the PS1 conformation. In this case, however, the R/G ratio returned to the baseline within 2 minutes, indicating that PS1 conformation was able to recover when the stimulant diffused (Fig. 1b). No change in the R/G ratio was observed in H_2_O-treated neurons or in neurons treated with Glu in the Ca^2+^/Mg^2+^ free media. Additional file 1 shows the GFP and RFP emission intensities and the R/G ratio in cells expressing GFP-PS1 (negative FRET control), GFP-RFP fusion (positive FRET control) or GFP-PS1-RFP, during 30 minutes recording. No GFP or RFP photobleaching was observed under the settings used. The increased R/G ratio after KCl application reflects increased FRET efficiency and is observed in G-PS1-R expressing cells only.

To verify that both treatments increase intracellular calcium load ([Ca^2+^]_I_), sister cultures were preloaded with Oregon Green 488 BAPTA-1 AM and imaged using time-lapse settings. Changes in the [Ca^2+^]_I_ strongly correlated with the R/G ratio (Fig. 1c).

These data reveal the dynamic nature of the PS1/γ-secretase and suggest that continuous insult (high intracellular Ca^2+^) maintains PS1/γ-secretase in a “closed” conformation, while a transient stressor modulates PS1 reversibly.

Furthermore, after 15 minutes of KCl treatment we could already detect increased levels of the secreted Aβ40 (150.5 ± 21.63 %, *p* = 0.0294) and Aβ42 (168.1 ± 27.24 %, *p* = 0.0286), resulting in an increased Aβ42/40 ratio (Fig. 1d) and consistent with the PS1 adopting a “closed” conformation at high intracellular Ca^2+^.

### Synaptotagmin 1 is a novel calcium-dependent PS1-binding partner

**Fig. 2:**
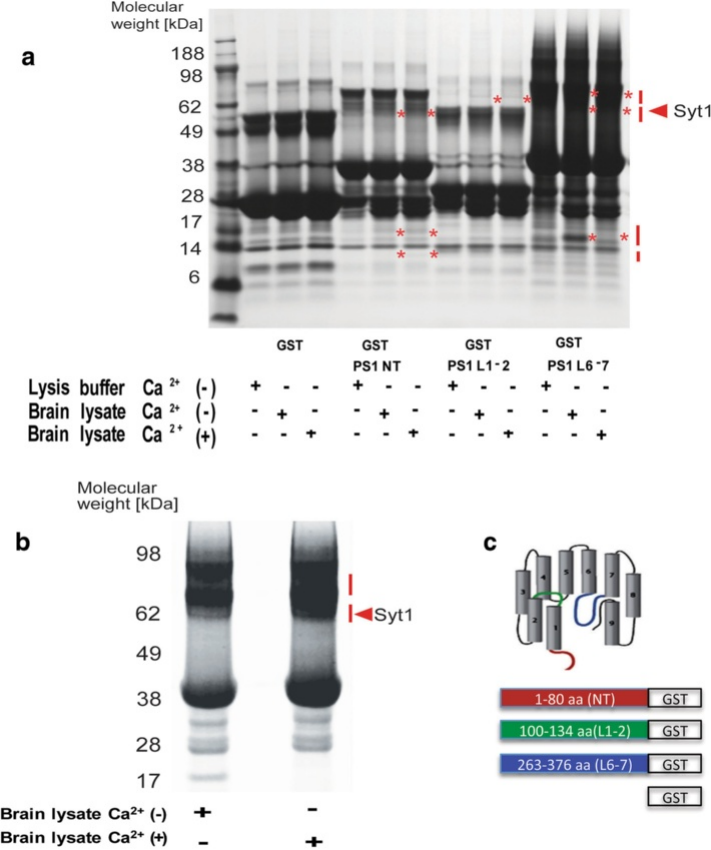
Mass spectrometry analysis of the PS1 interacting proteins. **a** Coomassie-stained gel of the proteins eluted from the column. The gel slices with bands of different sizes were excised from the Ca^2+^ (+) and Ca^2+^ (-) condition from the GST-PS1 pull-downs, and were sent for mass spectrometry analysis (indicated by lines to the *right* of the gel). The slice selection was based on the differences in the protein profiles/band intensities between GST-control and GST-PS1 pull-down, GST-PS1 pull-down from brain lysates vs. lysis buffer, and between Ca^2+^ (+) and Ca^2+^ (-) condition (these bands are indicated with the *asterisks*). The bands containing Syt1 are indicated with *arrowheads*. Three independent MS screens were performed. The Table shows number of peptides identified and the sequence coverage for Syt1 in Ca^2+^ (-) and Ca^2+^ (+) conditions; catenin1 delta is shown as Ca^2+^-independent control. **b** Image of the coomassie stained gel from a different MS experiment shows differences in the band intensities for proteins pulled down with the GST-PS1 L6-7 (the area is overexposed in Fig. 2a). The *arrowhead* points to the band containing Syt1. **c** Schematic representation of the PS1 molecule and the GST-fusion peptides used in the MS screen of the Triton X-100-digested mouse brain lysates for PS1-binding partners; *gray cylinders* correspond to PS1 transmembrane domains. PS1 fragments used in the pull-down are labeled in red, green and blue. *PS1* presenilin 1, *GST* glutathione S-transferase, *Syt1* synaptotagmin 1, *MS* mass spectrometry

To search for potential Ca^2+^-dependent modulators of the PS1/γ-secretase at the synapse, we performed an unbiased mass spectrometry (MS) proteomics screen of wild type (wt) mouse brain lysed in 1 % Triton X-100 in the presence or absence of Ca^2+^. Using GST-tagged peptides corresponding to PS1 domains or GST alone as a control, synaptotagmin 1 (Syt1) was identified as a strong candidate for novel PS1-interacting presynaptic protein (Table 1, Fig. 2, and Additional file 2). Syt1 interaction with PS1 was the strongest when GST-fused PS1 L6-7 peptide was used as “bait”, although a smaller number of Syt1 peptides was also pulled down with the GST-PS1 NT peptide. Since both L6-7 and PS1 NT sequences used for the pull down are localized within the post-endoproteolytic PS1 N-terminal fragment, the data suggest that Syt1 interacts with the PS1 NTF. Importantly, Ca^2+^ level affected the interaction between PS1 and Syt1, with robust PS1-Syt1 binding observed in the presence of high Ca^2+^. Identification of known Ca^2+^-insensitive PS1 interactors, such as catenin delta1, reaffirmed the specificity of the assay for Ca^2+^-dependent PS1 binding partners.

**Table 1:** Mass spectrometry screen identified Syt1 as a novel synaptic PS1-binding protein that shows calcium-dependent profile of the interaction

PS1 bait	Binding protein	Accession number	Gene symbol	Unique/total peptides		Sequence coverage		Protein function
				Ca^2+^(+)	Ca^2+^(-)	Ca^2+^(+)	Ca^2+^(-)	Ca^2+^(+)
NT	Synaptotagmin 1	NP033332	SYT1	4/4	None	11.6 %	None	Calcium sensor for synaptic vesicle fusion and neurotransmitter release
L6-7	Synaptotagmin 1	NP033332	SYT1	16/26	2/2	32.1 %	5.0 %	
L6-7	Catenin delta 1	NP031641	CTNND1	17/20	25/35	20.9 %	30.8 %	Known presenilin 1 interactor in the brain

The number of peptides identified and the sequence coverage for Syt1 in Ca^2+^ (-) and Ca^2+^ (+) conditions is shown. A known PS1 interacting protein, Catenin1 delta, is shown as Ca^2+^-independent control

### PS1 co-localizes with Syt1 at presynaptic terminals

To establish if PS1 co-localizes with Syt1 in the presynaptic terminals, we triple-immunostained synaptoneurosomes (SNSs) isolated from wt mouse cerebral cortex for: 1) PS1, 2) Syt1, and 3) either synapsin 1 (Syn1) and vesicular glutamate transporter 1 (vGlut1) or microtubule-associated protein 2 (MAP2), as pre- and postsynaptic markers, respectively.

**Fig. 3:**
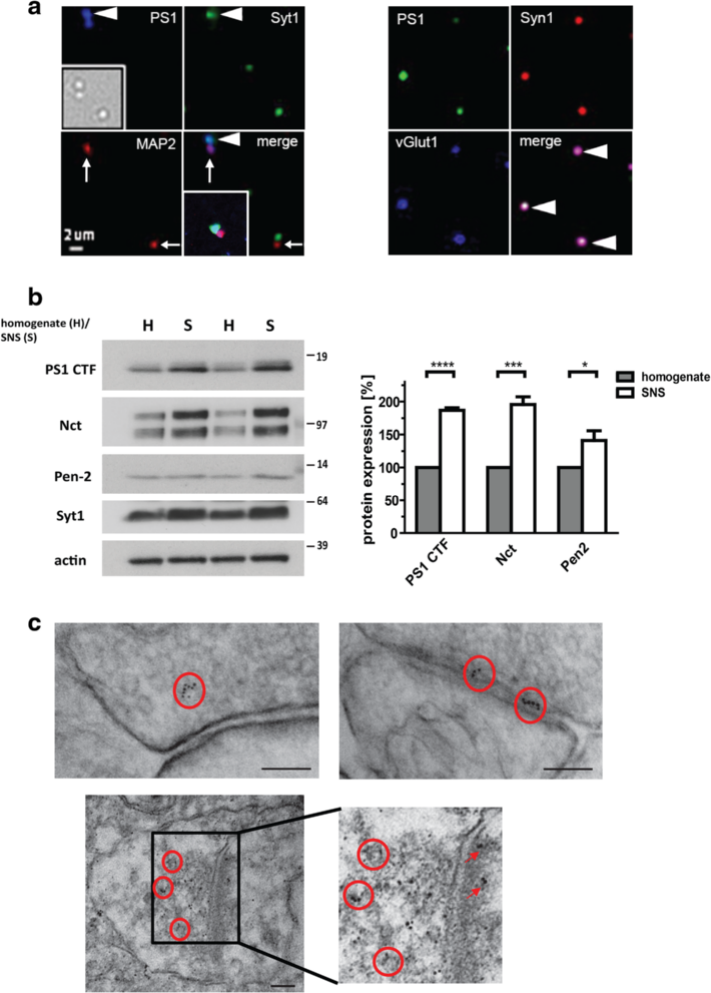
PS1/γ-secretase present in the pre- and post-synaptic terminals. **a** Confocal microscopy imaging shows co-localization of PS1 with Syt1 (left) and with synapsin1 and vGlut1 (right) in presynaptic compartments of isolated synaptoneurosomes (SNSs, *arrowheads*). *Arrows* show MAP2-positive post-synaptic buttons. *Insert* on the left shows a bright field image of isolated SNS. **b** Western blot analysis of PS1 CTF, Nct, Pen-2, Syt1 and β actin as a control in total homogenate (H) and synaptoneurosome (S) fractions from wild type mouse brain. Enrichment of the γ-secretase components in SNS vs. total brain homogenate is quantified. Data are presented as mean ± SEM, *n* = 4. Statistical significance was determined using the unpaired student *t*-test; * *p* < 0.05, *** *p* < 0.001. **c** Electron micrographs of synaptic terminals from mouse cortex immunostained for PS1 (gold particles). *Red circles* and *arrows* indicate positive staining in presynaptic and postsynaptic compartments, respectively. *PS1* presenilin 1, *Syt1* synaptotagmin 1, *vGlut1* vesicular glutamate transporter 1, *MAP2* microtubule-associated protein 2, *CTF* C-terminal fragment, *Nct* nicastrin, *Pen-2* presenilin enhancer 2

Bright field imaging and immunostaining demonstrate the presence of the snowman-shaped SNSs formed by pre- and post-synaptic terminals (inserts in Fig. 3a) and co-localization of endogenous PS1 with Syt1, Syn1, and vGlut1 at the presynapse, in addition to PS1 presence at the postsynapse (Fig. 3a). Of note, a fraction of the synaptic terminals was PS1 negative in both pre- and post-synaptic compartments. Enrichment of the PS1/γ-secretase in the SNSs was further confirmed by western blotting and PS1-colloidal gold immunoelectron microscopy of mouse brain tissue (Fig. 3b and c). The latter showed that PS1 is present in synaptic vesicles of both the reserve and release-ready pools at the presynaptic membrane and within the postsynaptic terminals.

### PS1 interacts with Syt1 on endogenous levels

**Fig. 4:**
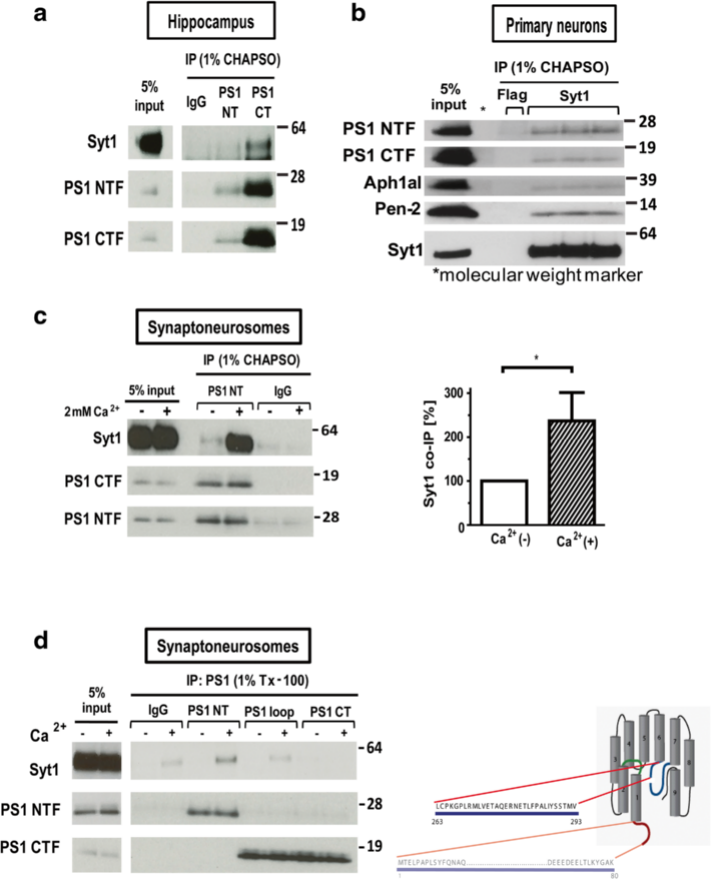
PS1 interacts with Syt1 on endogenous level. **a** Syt1 co-immunoprecipitates with PS1 from mouse hippocampi. The co-IP assay was conducted using anti-PS1 CT and anti-PS1 NT antibodies for pull-down; the detection antibodies are indicated to the *left* of each blot, *n* = 3. **b** PS1 co-immunoprecipitates with Syt1 from mouse cortical primary neurons. The co-IP assay was conducted using anti-Syt1 antibody for pull-down; the detection antibodies are indicated to the *left* of the blot. Other γ-secretase components pulled-down with Syt1 in the 1 % CHAPSO buffer are shown, *n* = 4. **c** Syt1 co-immunoprecipitates with PS1 from synaptoneurosome (SNS) fractions in a Ca^2+^-dependent manner. SNS were solubilized in 1 % CHAPSO buffer in the presence or absence of 2 mM CaCl_2_. N-terminal PS1 antibody (or IgG control) were used for pull-co-immunoprecipitated with PS1 NTF (normalized to the respective PS1 NTF band intensities). All the data are presented as mean ± SEM, *n* = 4. Statistical significance was determined using the unpaired student *t*-test, * *p* < 0.05. **d** Syt1 co-immunoprecipitates with PS1 from mouse SNSs solubilized in 1 % TritonX-100 buffer in the presence of 2 mM CaCl_2_ when anti-PS1 NT, but not anti-PS1 loop or anti-PS1 CT antibody, is used for pull-down. The detection antibodies are indicated on the left side of the blot. Schematic representation of the PS1 molecule; the presumed Syt1 interaction sites are shown. *PS1* presenilin 1, *Syt1* synaptotagmin 1, *IP* immunoprecipitation, *CT* C-terminus, *NT* N-terminus, *NTF* N-terminal fragment

The use of recombinant GST-PS1 peptide pull-down in the mass spectrometry screen has some limitations due to the possibility of improper folding. Therefore, to verify occurrence of the interaction on endogenous level, we employed a different approach: co-immunoprecipitation (co-IP) of PS1/γ-secretase with Syt1 using 1 % CHAPSO-solubilized extracts of mouse hippocampi, primary neurons, and cerebral cortex synaptoneurosomes (SNSs). Indeed, Syt1 was co-immunoprecipitated with PS1 (Fig. 4), suggesting a physiological function of the interactions between PS1 and Syt1 in the brain. Of note, other members of the γ-secretase complex were also pulled down with Syt1 from the 1 % CHAPSO-solubilized extracts, in which interactions among components of the γ-secretase complex are retained (Fig. 4a).

To validate the Ca^2+^-dependence of the interactions, SNS fractions were subjected to IP in the presence of 2 mM CaCl_2_ (Ca^2+^+) or 2 mM EGTA (Ca^2+^-). Indeed, more Syt1 was co-immunoprecipitated with PS1 in the high Ca^2+^ condition (Fig. 4c). There was no significant effect of Ca^2+^ on the immunoreactivity of total lysates (input lanes in Fig. 4c) or the efficiency of PS1 NTF and CTF interaction.

To examine which fragment of PS1 (NTF or CTF) predominantly binds Syt1, SNS fractions were lysed in 1 % Tx-100 buffer, disrupting interactions between the γ-secretase components [26]. The Syt1 band was detected only when anti-PS1 NT antibody was used for the pull-down, suggesting that Ca^2+^-bound Syt1 selectively interacts with the PS1 N-terminal fragment (Fig. 4d). Since the largest amount of the Syt1 peptides during the MS screen was pulled down with the PS1 peptide corresponding to the amino acids (aa) 263–376 of the L6–7 domain, the region was further narrowed to the aa 263–293, the C-terminal part of the PS1 NTF. Of note, a smaller number of the Syt1 peptides was also detected in the GST-PS1 NT (aa 1–80) pull-down; hence, it is possible that this region provides an additional, lower-affinity interface for the interaction (Fig. 4d).

**Fig. 5:**
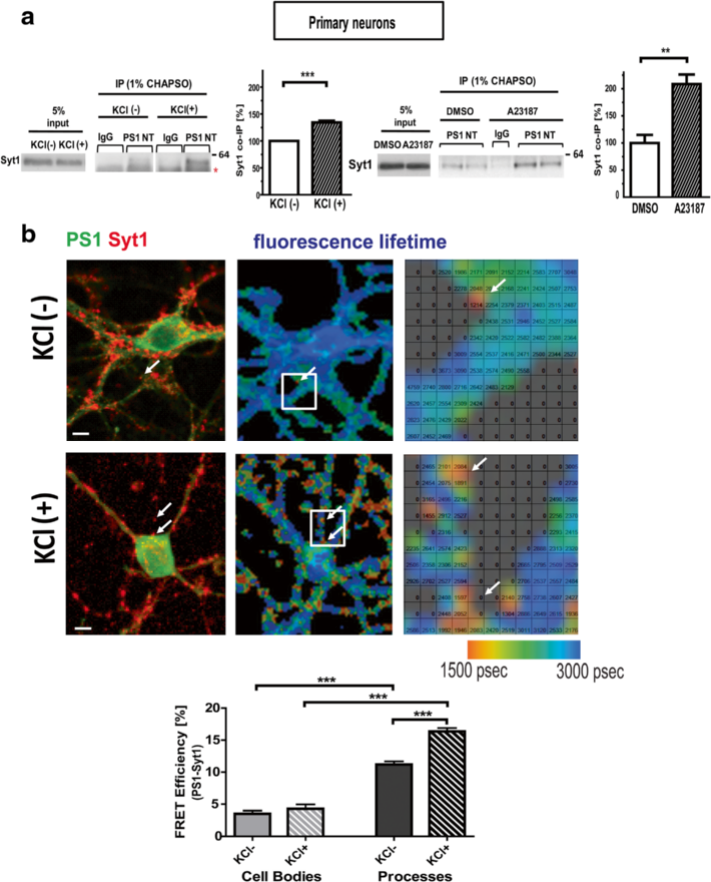
PS1 interacts with Syt1 in Ca^2+^-dependent manner in primary neurons. **a** Co-immunoprecipitation experiments demonstrate that KCl (*n* = 3) or calcium ionophore A23187 treatment (*n* = 3) of primary neurons that trigger Ca^2+^ influx strengthen the interaction between PS1 and Syt1. The *asterisk* shows a non-specific, heavy chain IgG band. The graph presents quantitative analysis of the Syt1 band intensity, mean ± SEM. Statistical significance was determined using the unpaired student *t*-test, ** *p* < 0.01, *** *p* < 0.001. **b** FLIM analysis of the PS1-Syt1 proximity in mouse cortical primary neurons treated for 5 minutes with 50 mM KCl (KCl+) or water control (KCl-). Fluorescence images show PS1 (green) and Syt1 (red) immunoreactivity. Scale bar: 5 μm. Pseudo-colored FLIM images depict lifetime of the Alexa 488 donor fluorophore. Colorimetric scale shows fluorescence lifetime in picoseconds. Zoomed *boxed* area shows FLIM image superimposed onto a table indicating average lifetimes for each pixel (~0.2 um^2^) of the image. Shortest lifetimes (yellow-to-red) reflect closest proximity between PS1 and Syt1. Bar graph presents [%] FRET efficiency (PS1-Syt1 proximity) recorded in the outlined regions of interest (ROIs) corresponding to the neuronal cell bodies or the processes. (mean ± SEM; *n* = 82 for cell bodies KCl(-), *n* = 63 for cell bodies KCl(+), *n* = 128 for processes KCl(-) and *n* = 146 for processes KCl(+); unpaired student *t*-test, *** *p* < 0.001). *PS1* presenilin 1, *Syt1* synaptotagmin 1, *FLIM* fluorescence lifetime imaging microscopy, *FRET* Förster Resonance Energy Transfer

Next, we tested whether Ca^2+^ influx into mouse primary neurons due to KCl-induced membrane depolarization or calcium ionophore treatment would enhance endogenous interaction between PS1 and Syt1. For this, 21–28 days in vitro cultured neurons were treated with KCl or Ca^2+^ ionophore (A23187) and subjected to co-IP/western blotting. An increased level of Syt1 co-immunoprecipitated with PS1 in the KCl-stimulated neurons (134.34 ± 3.14 %, *p* = 0.0004), compared to those treated with vehicle (Fig. 5a). An even higher increase was detected when a stronger stimulant, Ca^2+^ ionophore, was used (208.6 ± 22.78 %, *p* = 0.0089). Collectively, these data provide strong evidence for endogenous, activity- and Ca^2+^ influx-regulated PS1-Syt1 interactions in neurons.

### Syt1-PS1 interaction in intact neurons is stimulated by KCl treatment

To further validate and characterize endogenous PS1-Syt1 interactions in intact neurons, we employed an antibody-based fluorescence lifetime imaging microscopy (FLIM) analysis. The FRET efficiency (%E_FRET_), reflecting relative proximity between the fluorophore-labeled PS1 L6-7 epitope and Syt1, was increased in neurons stimulated with KCl, compared to vehicle-treated, suggesting more PS1-Syt1 interactions in the former (Fig. 5b).

To determine subcellular localization of the PS1 and Syt1 interactions, donor lifetime was color-coded and mapped on a pixel-by-pixel basis through the entire image. Shortest lifetimes (yellow-to-red pixels) were recorded mainly along the processes and co-localized with the dotted pattern of Syt1 immunoreactivity, presenting synaptic boutons as sites of the KCl-induced PS1-Syt1 interaction (Fig. 5b). Of note, KCl treatment resulted in a significant increase in the %E_FRET_ along the processes, without having a considerable effect on PS1-Syt1 interactions in the cell bodies. These findings reaffirm that PS1 and Syt1 interaction occurs on endogenous level, and that Ca^2+^-influx induces the interaction primarily at the Syt1-positive loci along the dendrites in primary neurons.

### Syt1 3D-A mutations impair Ca^2+^-dependent interactions between PS1 and Syt1

**Fig. 6:**
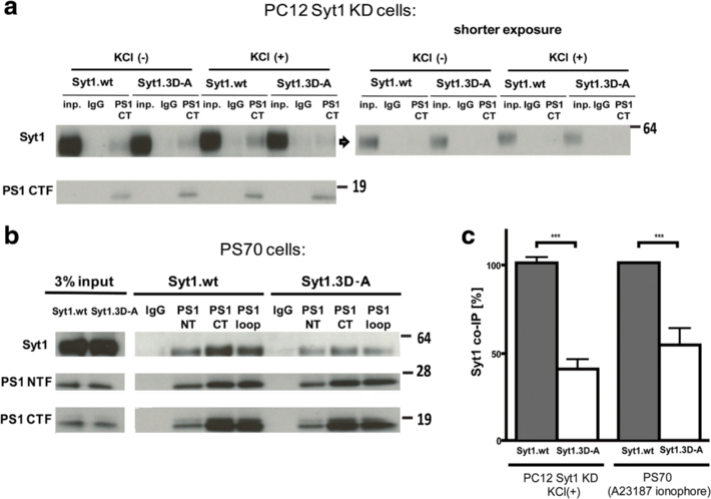
Mutations in Syt1 at Ca^2+^–binding aspartate residues disrupt Ca^2+^-dependent Syt1-PS1 interaction. **a** Western blot analysis of the Syt1 co-immunoprecipitated with PS1 from 1 % CHAPSO lysed Syt1 KD PC12 cells transiently transfected with Syt1.wt or Syt1.3D-A encoding vectors, following 15-minute 50 mM KCl (KCl+) or water control (KCl-) treatment. Inp.- input cell lysate shows a comparable level of Syt1 in cells transfected with wild type and 3D-A mutant Syt1. IgG (control) or PS1 CT antibody was used for pull-down. **b** PS70 CHO cells were transfected with Syt1.wt or Syt1.3D-A expression plasmids, treated for 15 minutes with 5 μM Ca^2+^ ionophore (A23187), and lysed in 1 % CHAPSO buffer. PS1-Syt1 complexes were immunoprecipitated using anti-PS1 NT, anti-PS1 CT or anti-PS1 loop antibodies. The immunoprecipitation efficiency was determined by re-probing the membrane with PS1 antibodies used for pull-down. **c** Quantitative analysis of the Syt1 co-immunoprecipitated with PS1 from PC12 (*n* = 3) and CHO (*n* = 6) in high calcium condition after KCl and calcium ionophore treatment, respectively. More Syt1.wt than Syt1.3D-A was co-immunoprecipitated with PS1 in the above conditions. All the data are presented as mean ± SEM. Statistical significance was determined using the unpaired student *t*-test, *** *p* < 0.001. *Syt1* synaptotagmin 1, *PS1* presenilin 1, *wt* wild type, *CT* C-terminus, *NT* N-terminus, *CHO* Chinese hamster ovary

To gain further insight into the Ca^2+^-dependency of the Syt1 interaction with PS1, we constructed a 3D-A Syt1 mutant by substituting for alanine three aspartate residues important for Ca^2+^ binding [27]. PC12 cells lacking Syt1 (Syt1 KD) were transfected with wild type or 3D-A Syt1-V5 plasmids, treated with KCl or vehicle, harvested, and subjected to co-IP/western blotting. As expected, significantly enhanced interaction of wt Syt1-V5 with PS1 was observed in response to KCl. In contrast, 3D-A Syt1-V5 binding to PS1 was significantly reduced compared to that of wt Syt1 in KCl-treated cells (Fig. 6a, c). Analogously, the interaction between mutant 3D-A Syt1-V5 and PS1 in Ca^2+^ ionophore-treated Chinese hamster ovary (CHO) cells was significantly weaker compared to that of wt Syt1-V5 and PS1 (Fig. 6b, c). These results support the importance of Ca^2+^ binding to Syt1 in the regulation of the PS1-Syt1 interactions.

### Syt1 affects endogenous Aβ production and PS1 conformation

**Fig. 7:**
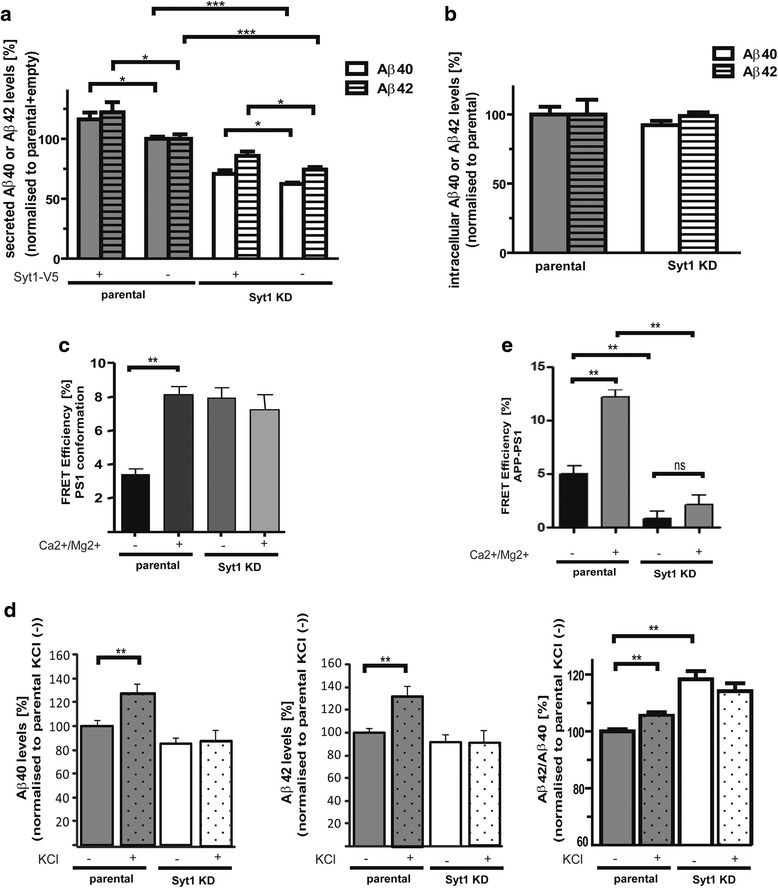
Aβ production/secretion and PS1 conformation are altered by Syt1 KD. **a**, **b** ELISA measurements of the endogenous (A) secreted and (B) intracellular Aβ40 and Aβ42 in parental and Syt1 KD PC12 cells stimulated for 15 minutes with 50 mM KCl. The secreted Aβ levels determined in pmol were normalized to the total protein extracted from the cells in the same well. (A) *n* = 5 for parental + Syt1-V5 and *n* = 6 for all other conditions; (B) *n* = 4. 100 % equals to 212.93 pmol/g and 20.06 pmol/g for secreted Aβ40 and Aβ42, respectively. 100 % for intracellular Aβ40 and Aβ42 equals to 1.92 pmol/g and 0.575 pmol/g, respectively. **c** FLIM analysis of the FRET efficiency [%] reflecting PS1 conformational changes in parental and Syt1 KD PC12 cells; *n* = 101 cells for parental Ca^2+^(-), *n* = 103 for parental Ca^2+^(+), *n* = 84 for Syt1 KD Ca^2+^(-) and *n* = 86 for Syt1 KD Ca^2+^(+). Higher values correspond to “closed” conformational state of PS1. The cells were placed in Ca^2+^/Mg^2+^-free (-) or Ca^2+^/Mg^2+^-containing (+) medium, prior to treatment with 50 mM KCl. **d** ELISA measurements of the endogenous Aβ40, Aβ42 and Aβ42/Aβ40 ratio in the conditioned medium from parental and Syt1 KD cells (*n* = 8). **e** FLIM analysis of endogenous APP and PS1 proximity (FRET efficiency [%]) in parental and Syt1 KD PC12 cells; *n* = 83 cells for parental Ca^2+^(-), *n* = 89 for parental Ca^2+^(+), *n* = 70 for Syt1 KD Ca^2+^(-) and *n* = 64 for Syt1 KD Ca^2+^(+). The cells were treated for 5 minutes with 50 mM KCl in Ca^2+^/Mg^2+^-free or normal medium, fixed, and immunostained for PS1 and APP. The data in all panels of the Figure are presented as mean ± SEM. The statistical significance was determined using the unpaired student *t*-test. *p < 0.05; ** *p* < 0.01; ***p < 0.001. *Aβ* amyloid β, *PS1* presenilin 1, *Syt1* synaptotagmin 1, *FLIM* fluorescence lifetime imaging microscopy, *FRET* Förster Resonance Energy Transfer, *APP* amyloid precursor protein

The above data provide strong evidence that Ca^2+^ influx promotes Syt1-PS1 interactions and dynamically modulates PS1 conformation in neurons. Therefore, we reasoned that Syt1 might be involved in regulation of the Aβ production/secretion and/or PS1 conformation. To gain insight into how Syt1 may modulate Aβ levels, we employed Syt1 knockdown and overexpression approaches using parental and Syt1 KD PC12 cells (Additional file 3). Aβ40 and Aβ42 levels were reduced by 37.69 ± 2.15 % and 25.63 ± 4.31 % (*p* < 0.0001), respectively, in Syt1 KD cells (Fig. 7a). The deficits were partially rescued by transient Syt1-V5 expression in Syt1 KD cells. Conversely, transient overexpression of Syt1-V5 in parental PC12 cells led to increased levels of both Aβ40 and Aβ42, by 16.30 ± 5.46 % and 22.16 ± 8.66 % (*p* < 0.05), respectively. Of note, the levels of intracellular Aβ40 and Aβ42 were not significantly different between the parental and Syt1 KD PC12 cells (Fig. 7b), suggesting that Aβ generation may be impaired.

To test whether KCl/Ca^2+^ influx-induced changes in PS1 conformation and Aβ production/secretion observed in neurons (Fig. 1b, d) are mediated by Syt1, we monitored PS1 NT-loop proximity and Aβ40 and Aβ42 levels in parental and Syt1 KD PC12 cells. The cells were transfected with G-PS1-R FRET reporter and treated with KCl in regular or Ca^2+^/Mg^2+^ free media. Similarly to neurons, a significant increase in the %E_FRET_ was observed in parental PC12 cells in the regular, Ca^2+^/Mg^2+^-containing (but not in the Ca^2+^/Mg^2+^-free) media, indicating that KCl-triggered pathological “closed” PS1 conformation requires the presence of Ca^2+^ ions (Fig. 7c). This corresponded to the KCl-induced increase in the Aβ40 and Aβ42 levels and the Aβ42/40 ratio in parental PC12 cells (Fig. 7d). On the contrary, no changes in PS1 conformation and Aβ in response to Ca^2+^ influx were detected in Syt1KD cells. Interestingly, whereas %E_FRET_ (Fig. 7c) and the Aβ42/40 ratio (Fig. 7d) were not affected by calcium in Syt1 KD, their baseline values were significantly higher in the absence of Syt1. These data suggest that, although the total level of Aβ is reduced by Syt1 KD, the presence of Syt1 is required for the maintenance of the Aβ42/40 ratio and PS1 in the physiological, “normal” conformation.

Since Aβ levels were significantly reduced in Syt1 KD cells, we tested whether interactions between APP substrate and PS1/γ-secretase may be affected. We detected decreased proximity between the PS1 loop domain and APP CT in Syt1 KD cells, suggesting reduced PS1-APP interaction in the absence of Syt1 (Fig. 7e). In addition, while KCl treatment of parental PC12 cells increased %E_FRET_ (PS1-APP proximity), it had no effect on the %E_FRET_ in Syt1 KD cells. Taken together, these data suggest that Syt1 may modulate Aβ levels by influencing the assembly and/or activity of PS1/γ-secretase and its interaction with the APP substrate.

### Syt1 modulates γ-secretase activity

**Fig. 8:**
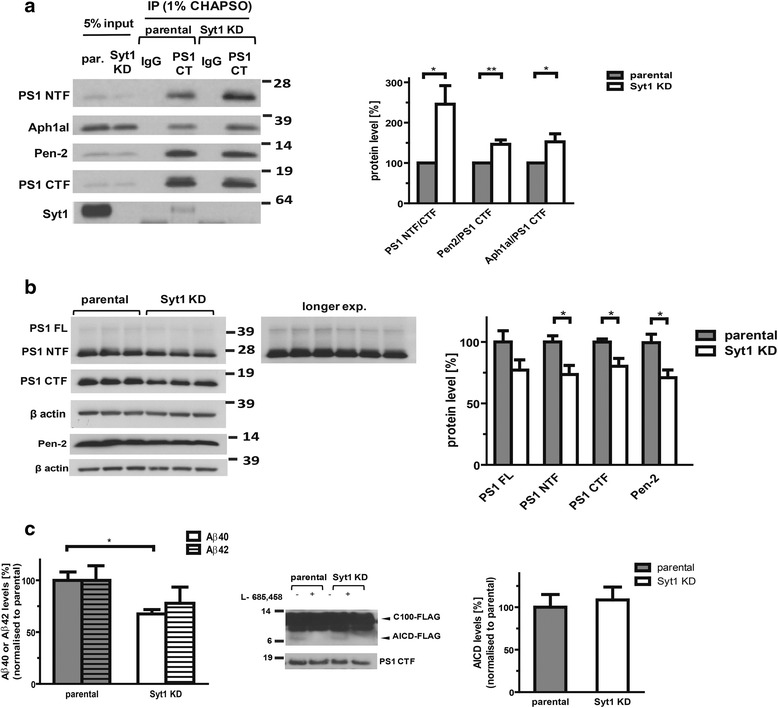
Syt1 influences the architecture of γ-secretase complex and Aβ generation but not AICD production. **a** Western blot analysis of the γ-secretase complex members co-immunoprecipitated from parental and Syt1 KD PC12 cells lysed in 1 % CHAPSO (*n* = 4). The *bottom* gel shows the level of Syt1 in the input lysates and after pull-down with PS1 C-terminal antibody or IgG control. The graph presents quantitative analysis of the PS1 NTF, Pen-2 and Aph1a co-IPed with the PS1 C-terminal fragments. The intensities of the bands were measured by densitometry, and adjusted to the intensity of the input bands and to the PS1 CTF (IP efficiency control). **b** Quantitative analysis of the PS1 FL, PS1 NTF, PS1 CTF, and Pen-2 in total protein extracts from parental and Syt1 KD PC12 cells (*n* = 4 for PS1 FL, *n* = 8 for PS1 NTF and PS1 CTF, *n* = 7 for Pen-2). The intensities of the bands were normalized to β actin levels. **c** Cell-free γ-secretase activity assay using parental and Syt1 KD PC12 cell membrane preparations and recombinant C100-FLAG as a substrate. The amounts of Aβ40 and Aβ42 were determined by sandwich ELISA and normalized to the levels of PS1 CTF in the corresponding samples. The intensities of AICD-FLAG bands were measured by densitometry and normalized to PS1 CTF. The quantitative data in all panels are presented as mean ± SEM, *n* = 4. Statistical significance was determined using the unpaired student *t*-test, * *p* < 0.05, ** *p* < 0.01. *Syt1* synaptotagmin, *Aβ* amyloid β, *AICD* APP intracellular domain, *APP* amyloid precursor protein, *PS1* presenilin 1, *NTF* N-terminal fragment, *Pen-2* presenilin enhancer 2, *IP* immunoprecipitation, *CTF* C-terminal fragment, *FL* full length

A co-IP/western-blot analysis of the PS1 CTF co-immunoprecipitation with PS1 NTF and with the other γ-secretase components in parental and Syt1 KD PC12 cells showed increased binding between PS1 CTF and NTF, as well as between PS1 and Pen-2 and Aph1a in the absence of Syt1 (Fig. 8a). This indicates that Syt1 deficiency affects γ-secretase architecture and promotes tighter association of its components. The finding is consistent with the more compact “closed” PS1 conformation in Syt1 KD cells, as detected by the FLIM assay. At the same time, decreased levels of the PS1 N-terminal and C-terminal fragments along with the diminished Pen-2 were detected in Syt1 KD cells (Fig. 8b), suggesting reduction of the functional γ-secretase in the absence of Syt1.

**Fig. 9:**
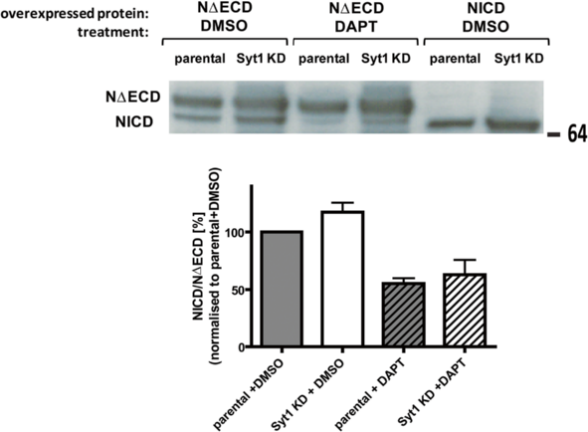
Notch1 intracellular domain (NICD) generation is not affected in Syt1 KD PC12 cells. Parental and Syt1 KD PC12 cells were transfected with Myc-tagged, constitutively active Notch1 with deleted extracellular domain (NΔECD), which is an immediate substrate of the γ-secretase. The cells were treated with DMSO or γ-secretase inhibitor — DAPT. Transfection with a plasmid encoding Notch1 intracellular domain (NICD) was used as a control. Western blot analysis shows no difference in the production of the Notch1 intracellular domain, as detected by anti-myc antibody, between parental and Syt1 KD PC12 cells. Data are presented as the NICD/NΔECD ratio; mean ± SEM, *n* = 4. Statistical significance was determined using unpaired student *t*-test. *Syt1* synaptotagmin 1

To test more directly if Syt1 may affect PS1/γ-secretase activity, we performed a cell-free in vitro γ-secretase activity assay using recombinant C100-FLAG substrate (APP β-CTF) and membrane preparations from parental and Syt1 KD PC12 cells containing active γ-secretase. A ~30 % reduction of the Aβ40 production was detected in Syt1 KD cells (Fig. 8c). A similar trend was recorded for Aβ42 but the data did not reach statistical significance. These data confirm partial loss of γ-secretase catalytic activity in the absence of Syt1. Of note, the APP intracellular domain (AICD) level remained unaltered (Fig. 8c), supporting the hypothesis that γ-secretase cleavage of the APP β-CTF occurs in a step-wise manner [28, 29], and loss of γ-secretase activity results in “incomplete digestion” of the APP CTF substrate in Syt1 KD cells. Likewise, the γ-secretase-dependent cleavage of Notch1 at the membrane-cytoplasm interface leading to generation of the Notch1 intracellular domain (NICD) was not affected in the absence of Syt1 in PC12 Syt1 KD cells (Fig. 9).

### Syt1 modulates APP processing

**Fig. 10:**
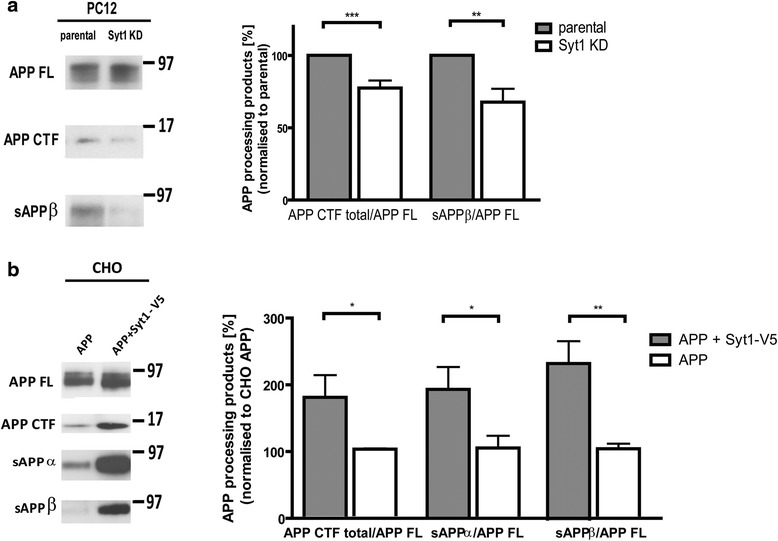
APP CTF and sAPPβ production are reduced in the absence of Syt1. Western blot analysis of the APP processing in PC12 (**a** *endogenous APP*) and CHO (**b** *stably overexpressed human APP*) cells. The levels of sAPPβ, sAPPα, and APP CTFs (total) were normalized to FL APP. Data are presented as mean ± SEM, (*A*) *n* = 6 for APP CTF and *n* = 8 for sAPPβ, (*B*) *n* = 7 for APP CTF, *n* = 6 for sAPPα, *n* = 5 for sAPPβ. Statistical significance was determined using the unpaired student *t*-test, * *p* < 0.05, ** *p* < 0.01, *** *p* < 0.001. *APP* amyloid precursor protein, *sAPPβ* secreted APPβ, *Syt1* synaptotagmin 1, *CHO* Chinese hamster ovary cells, *CTF* C-terminal fragment, FL full length

The role of Syt1 in APP processing was further investigated by analyzing the levels of endogenous APP C-terminal fragments and secreted APPβ (sAPPβ) in the presence and absence of Syt1 in PC12 cells. About a 22.57 ± 5.21 % (*p* < 0.001) decrease in the total APP CTF/FL ratio was observed in Syt1 KD cells, suggesting that α- and/or β-secretase cleavage of APP may be affected (Fig. 10a). Indeed, the sAPPβ/APP FL ratio was decreased by about 32.25 ± 9.15 % (*p* < 0.001) in the absence of Syt1 (Fig. 10a). On the other hand, in CHO cells lacking endogenous Syt1 but stably overexpressing human APP and Syt1-V5 or only APP, the APP CTF/APP FL, sAPPα/APP FL, and sAPPβ/APP FL ratios were significantly increased in cells overexpressing Syt1 (Fig. 10b).

### BACE1 maturation and degradation are affected in Syt1 KD cells

**Fig. 11:**
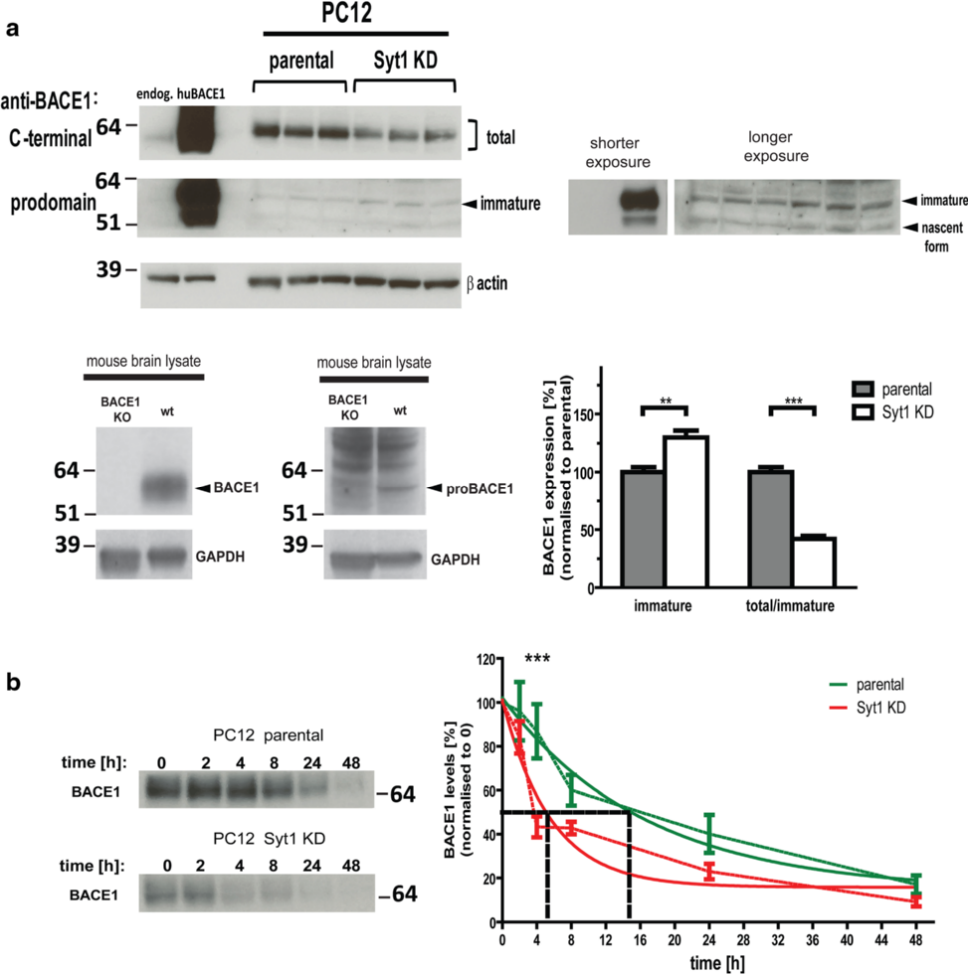
Syt1 modulates BACE1 maturation and stability. **a** Levels of total and immature BACE1 in protein lysates from parental and Syt1 KD PC12 cells were determined by western blotting using anti-BACE1 antibodies directed against the C-terminus or prodomain of BACE1, respectively (*n* = 5). The specificity of the antibodies was confirmed using overexpression of human BACE1 in CHO cells (*top gel*) and brain lysates from BACE1 KO and control littermate mice (*bottom gel*). The intensities of all BACE1 bands were quantified by densitometry and normalized to the levels of β actin. Data are presented as mean ± SEM. Statistical significance was determined using unpaired student *t*-test. ** *p* < 0.01, *** *p* < 0.001. **b** Analysis of BACE1 degradation. Parental and Syt1 KD PC12 cells were treated with cycloheximide and harvested 0, 2, 4, 8, 24, and 48 hours after the treatment (*n* = 5). Western blot presents BACE1 expression over time. Anti-BACE1 C-terminal antibody was used for detection. The quantitative analysis of BACE1 levels revealed a significantly reduced half-life of BACE1 in Syt1 KD PC12 cells compared to the parental PC12 control (marked with *black dashed lines*). Statistical significance was determined using 2-way ANOVA with Bonferroni post-test, *** *p* < 0.001

The reduced amount of sAPPβ suggests that in addition to the γ-secretase activity, BACE1 activity may also be affected in Syt1 KD cells. Indeed, the level of endogenous total BACE1 was significantly decreased, whereas the level of immature BACE1 detected by prodomain-specific antibody was increased in Syt1 KD cells, relative to that in parental PC12 cells. Thus, the total/immature BACE1 ratio was reduced by 58.19 ± 5.14 % (*p* < 0.0001) in the absence of Syt1 (Fig. 11a).

The low levels of mature BACE1 were at least partially due to a drastic reduction in the BACE1 protein half-life in Syt1 KD cells, as revealed by the cycloheximide pulse chase assay (Fig. 11b). Of note, no difference in the stability of PS1 CTF and APP between parental and Syt1 KD cells was observed (Additional file 4).

### Syt1 affects localization of APP processing enzymes

**Fig. 12:**
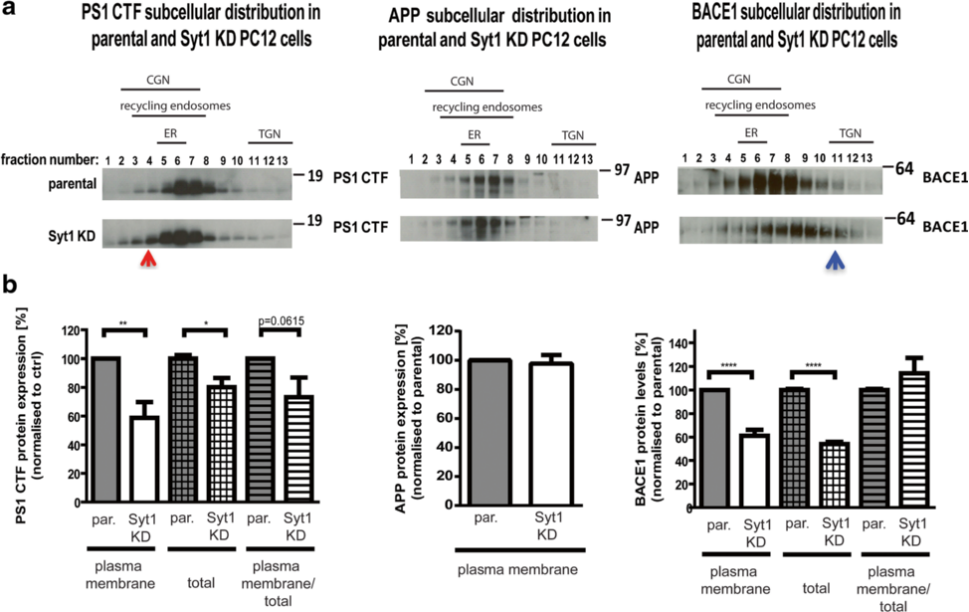
Subcellular compartmentalization of PS1, APP, and BACE1 in the presence and absence of Syt1. **a** Western blots show distribution of PS1, APP, and BACE1 in 13 subcellular fractions from parental and Syt1 KD cells. The arrows indicate a shift in the subcellular distribution in Syt1 KD cells. The enrichment of the respective subcellular compartments was determined by western blotting with anti-calreticulin, anti-GM130, anti-Tgn46, and anti-Rab11 antibodies. The fractions that correspond to the respective intracellular compartments are indicated *above* the western blots. **b** Bar graphs present quantitative analysis of the PS1, APP, and BACE1 levels at the plasma membrane, in total protein lysates, and the ratio of plasma membrane/total protein. Data are presented as mean ± SEM, *n* = 4 for PS1 in the membrane fraction, *n* = 8 for PS1 in total lysate, *n* = 5 for APP, and *n* = 5 for BACE1. Statistical significance was determined using the unpaired student *t*-test, * *p* < 0.05, ** *p* < 0.01; ***p < 0.001. *PS1* presenilin 1, *APP* amyloid precursor protein, *BACE1* β-secretase 1, *Syt1* synaptotagmin 1

To determine whether Syt1 may modulate Aβ production by influencing trafficking of BACE1, PS1/γ-secretase, or APP, we analyzed their distribution in the parental and Syt1 KD PC12 cells using subcellular fractionation (Fig. 12a). We found that lack of Syt1 affects trafficking of PS1 CTF and BACE1 but not APP. The PS1 CTF has been retained within the earlier secretory compartments in Syt1 KD cells. On the other hand, the distribution of BACE1 has shifted towards the trans-Golgi network in the absence of Syt1.

The cell-surface biotinylation approach revealed a significantly reduced amount of BACE1 and PS1 CTF on the surface of Syt1 KD cells as compared to parental PC12 cells (Fig. 12b). However, since the levels of mature, functionally active γ-secretase (PS1 CTF/NTF) and BACE1 were considerably reduced in the absence of Syt1, we calculated the ratio of plasma membrane to total level for each of the proteins. The adjusted values were not significantly different between the cell lines, although there was a trend towards reduced PS1 CTF plasma membrane/total ratio in Syt1 KD cells, suggesting that the impaired intracellular transport of PS1 might cause this slight reduction of the PS1/γ-secretase at the cell surface.

To determine if the observed mislocalization of PS1 and BACE1 in the absence of Syt1 was due to a broader effect of the Syt1 KD on protein trafficking, we analyzed subcellular distribution of another synaptic protein, synaptophysin (Syp), as well as other γ-secretase members/transmembrane proteins, Nct and Pen-2, in Syt1 KD and parental PC12 cell lines (Additional file 5A and B bottom panel). No major differences in the abundance of the target proteins within the respective fractions were observed between the cell lines. Furthermore, the overall transport fidelity was not noticeably altered by the lack of Syt1, as shown by comparable distribution of the compartment specific markers within the respective fractions in PC12 cells with and without Syt1 (Additional file 5B). This further reaffirms that Syt1 selectively affects PS1 and BACE1, and that their misdistribution is not due to impairments in the overall intracellular transport fidelity in the Syt1 KD cells.

### Syt1 is unlikely a substrate for PS1/γ-secretase

Since Syt1 has a relatively short N terminus, single-pass transmembrane domain and a larger cytoplasmic region, a structure similar to the previously known PS1/γ-secretase substrates, we tested if Syt1 may be a novel γ-secretase substrate. There was no difference in the levels of Syt1 and its proteolytic fragments between DMSO- and γ-secretase inhibitors-treated primary neurons and mouse embryonic fibroblasts (MEF), or in MEFs lacking PS1/PS2 (Additional file 6). Hence, it is unlikely that Syt1 is a PS1/γ-secretase substrate.

## Discussion

Synaptotagmin 1 (Syt1), a synaptic vesicle associated protein, was identified as a novel Ca^2+^-dependent binding partner of presenilin 1 (PS1). Moreover, a new role of Syt1 in modulation of the PS1 conformation and Aβ production via several mechanisms was revealed.

The Ca^2+^-dependent profile of PS1 binding to Syt1 suggests that Ca^2+^ may function as a switch controlling PS1 interaction with the synaptic vesicle (SV) protein intimately involved in SV exocytosis and neurotransmitter release. An involvement of presenilins in the activity-dependent regulation of neurotransmission was shown by Zhang et al. [30], reporting Ca^2+^-linked impairment of synaptic facilitation and neurotransmitter release as a result of selective pre- but not post-synaptic inactivation of PS1/2. Together, these findings suggest that PS1 may be involved in regulation of the neurotransmitter release via Ca^2+^-triggered interaction with Syt1. In addition, our study shows that since presynaptic terminals are the major sites of the Ca^2+^-induced interaction in neurons, as determined by FLIM (Fig. 5b), Syt1 might play a role in modulation of the Aβ production/secretion locally at the synaptic terminals. This does not, however, exclude the role of Syt1 in Aβ production away from the synapse and/or in a PS1-independent manner.

Synaptic activity and Ca^2+^ flux are tightly linked to Aβ production [11–13, 31] but the molecular mechanisms underlying modulation of the Aβ production at the synapse remain elusive. A sustained release of the neurotoxic Aβ42 species in response to stimulation of isolated nerve terminals [21], and deposition of oligomeric Aβ at the excitatory synapses in the brain were reported [32, 33]. The formation of Aβ oligomers and their neurotoxicity depends not only on the increased amount of Aβ but more importantly on the Aβ42/40 ratio [34]. Familiar AD causing mutations in PS1, PS2, or APP consistently increase the relative ratio between the Aβ42 and Aβ40 peptides, irrespective of the individual levels of Aβ42 and Aβ40 [35–37]. We have previously reported that increased Aβ42/40 ratio is tightly linked to pathogenic “closed” conformation of PS1 [22, 23, 25, 38]. Concomitant with these findings, our current study demonstrates that continuous neuronal activation and elevated intracellular calcium induce pathogenic “closed” conformation of PS1, and provide a mechanistic explanation of the observed targeted accumulation of Aβ oligomers at the synapse.

Structural changes in PS1 are observed during normal aging, they precede Aβ plaque deposition, and are exacerbated in sporadic AD brains [39]. We propose that PS1/γ-secretase exists in a dynamic equilibrium of different conformational states, which we call “closed” and “open” based on the FRET measurements, that may modulate PS1 function [23, 25]. Although it is possible that familial AD (fAD) mutations in PS1 [23] or recruitment of Aph1B isoform [38] produce more stable γ-secretase complexes with PS1 in pathogenic “closed” conformation, our current study shows that allosteric changes in the pre-existing γ-secretase complexes can occur in response to Ca^2+^. Importantly, these changes are reversible, as shown after a brief puff of glutamate, and closely correlate with the cytosolic Ca^2+^ level. We demonstrate that in addition to promoting “closed” PS1 conformation, prolonged depolarization of neurons and continuing high intracellular Ca^2+^ also increased the level of both Aβ40 and Aβ42 and, importantly, led to an increased Aβ42/40 ratio. These data are consistent with Kim et al. [21] reporting a similar increase in Aβ42 levels after KCl-induced depolarization of isolated nerve terminals, synaptoneurosomes, and are in line with the early detection of Aβ deposits in the default mode network of asymptomatic older adults [10]. Interestingly, a spike-burst stimulation of hippocampal neurons increased release of only Aβ40 but not Aβ42, accompanied by PS1 conformational changes consistent with reduced FRET efficiency [40]. These data suggest that diverse stimuli and/or their duration may have different outcomes, with continued activation and/or persistent high calcium inducing pathogenic change in PS1/γ-secretase and elevated Aβ42/40 ratio that would ultimately lead to Aβ deposition in the brain.

Our current study reveals that Syt1 may be required for the maintenance of the physiological “open” PS1 conformation, as pathogenic conformational change of PS1 was detected in the absence of Syt1. Consistent with the PS1 conformational change, the ratio of Aβ42 to Aβ40 was elevated in Syt1 KD cells, although the level of both Aβ40 and Aβ42 was significantly reduced in the absence of endogenous Syt1 and increased by transient Syt1-V5 overexpression. This is consistent with our findings in stably Syt1 transfected PC12 and CHO cells [41]. Interestingly, whereas KCl treatment induced PS1 conformational change and triggered Aβ production in neurons and parental PC12 cells, it had no effect on either in Syt1 KD cells. Thus, it is highly likely that in addition to its role in exocytosis, Syt1, presenting a strong and specific synaptic expression pattern in neurons, may also modulate Aβ production/secretion locally at the synaptic terminals.

We identified several molecular mechanisms that underlie Syt1-mediated regulation of Aβ, both PS1 interaction-dependent and independent. Syt1 and its interaction with PS1 may be needed for maintaining PS1 in the “open” conformation, and modulation of specific Aβ species produced. We observed tighter association of the γ-secretase components (consistent with the “closed” PS1 conformation), diminished amount of PS1 NTF/CTF, and reduced γ-secretase activity in Syt1 KD compared to parental cells, indicating that architecture and activity of the γ-secretase complex are impaired in the absence of Syt1. Importantly, whereas Aβ production was reduced by Syt1 KD, the PS1/γ-secretase dependent epsilon and S3 cleavages, responsible for AICD and NICD generation, respectively, were not affected. This finding is consistent with the step-wise γ-secretase cleavage model that proposes a three amino acid spaced cleavage mechanism for γ-secretase mediated intramembrane proteolysis [28, 29, 42]. The effect of Syt1 KD is also in agreement with the proposed γ-secretase loss-of-function due to familial AD mutations in PS1, which also display “closed” conformation and biochemically result in a partial loss of the γ-secretase activity due to inability of ‘complete digestion’ of the APP substrate, generating fewer but longer Aβs (for review see [37]).

Calcium binding to Syt1 activates SV release machinery [18] and, hence, may promote exocytosis of Aβ at the synapse. Indeed, active PS1/γ-secretase complex, APP and BACE1 were found in SVs enabling Aβ production within the vesicles ([43–47] and this study).

Moreover, we found that Syt1 knockdown selectively impairs intracellular trafficking of PS1 and BACE1 and, thus, may affect Aβ via trafficking abnormalities and away from the synapse. Syt1 is also a direct binding partner of APP [41, 48] and, therefore, may regulate APP processing by acting as a chaperon for APP and/or PS1, influencing their subcellular localization, alignment and interaction. Indeed, our FLIM assay revealed diminished APP-PS1 proximity in the absence of Syt1, which could be due to observed mislocalization of PS1 in the Syt1 KD cells. Finally, we demonstrate that Syt1 expression is crucial for localization, maturation, and stability of BACE1; thus, it may also modulate earlier steps of the APP processing. We found that the subcellular localization of BACE1 but not APP is altered in the absence of Syt1. This is consistent with other studies showing that trafficking of APP and BACE1, namely surface-to-endosome transport and sorting, could be distinctly regulated within the cell [49–51]. It should also be mentioned that since PS1 is involved in BACE1 maturation [52], there is a possibility that affected PS1 function in Syt1 KD has a downstream effect on BACE1.

Taken together these findings provide compelling evidence supporting a role of Syt1 in Aβ production. Namely, in addition to facilitation of the Aβ secretion via exocytosis, Syt1 plays a direct role in the activity-mediated regulation of APP processing by: (1) modulating PS1/γ-secretase conformation, catalytic activity, and interactions with APP; (2) affecting BACE1 maturation and stability; and (3) mediating BACE1 and PS1 subcellular compartmentalization.

The discovery of the novel Ca^2+^-dependent interactions between PS1 and Syt1 links several well-established players implicated in AD pathogenesis, such as elevated Ca^2+^, structural changes in PS1/γ-secretase, and toxic Aβ42 specie generation with synaptic proteins critical for SV/neurotransmitter release. It also provides strong evidence for Syt1 being a Ca^2+^-sensitive modulator of the PS1/γ-secretase, APP processing, and consequent Aβ production and deposition at the pre-synapse, suggesting a new role for Syt1 in neurodegeneration. Lack of the Syt1 effect on Notch1 ICD generation may encourage novel synapse-specific therapeutic strategies targeting Syt1-PS1 interaction.

## Conclusions

Synaptic loss caused by local accumulation of the oligomeric amyloid β (Aβ) is the strongest and the earliest correlate of Alzheimer’s disease dementia. However, events and proteins that influence synaptic Aβ accumulation are poorly understood. In a search for early modifiers of the synaptic Aβ we identified synaptic protein, synaptotagmin 1 (Syt1), as a novel calcium-regulated interactor of the Aβ cleaving enzyme, presenilin 1 (PS1)/γ-secretase. We found that Syt1 may function as activity-dependent regulator of the synaptic Aβ by modulating PS1 catalytic activity and conformational state, BACE1 maturation and stability. These novel discoveries provide a mechanistic link between major players implicated in AD pathogenesis: high calcium, toxic Aβ42 specie, PS1/γ-secretase conformational changes, and synapse. Furthermore, these findings may open avenues for new, synapse-targeting therapeutic strategies to halt the Alzheimer’s disease progression.

## Methods

### Expression plasmids

The GFP-PS1-RFP (G-PS1-R) construct encoding wild type (wt) human presenilin 1 (PS1) with enhanced green fluorescent protein (EGFP) fused to the N-terminus and red fluorescent protein (RFP) inserted into the loop domain of PS1 was used for the fluorescence life time imaging (FLIM) assay of PS1 conformational changes [25]. The constructs with EGFP fused to the N-terminus of PS1 (G-PS1), RFP inserted into the loop domain of PS1 (PS1-R), and RFP-GFP fusion (R-G fusion) plasmid, in which RFP is fused to the N-terminus of EGFP with a short linker, were used as controls.

Human wt synaptotagmin 1 (Syt1) was cloned into pcDNA™ 6 V5 Myc expression vector (Life Technologies, Grand Island, NY). Mutations that affect calcium binding to Syt1 (D230A, D309A, and D365A) were introduced using a QuickChange site-directed mutagenesis kit (Stratagene, La Jolla, CA) to generate Syt1 3D-A plasmid. The empty pcDNA™ 6 V5 Myc vector was used as a control.

pCS2 Notch1 ΔEC-6MT vector encoding C-terminally Myc-tagged and N-terminally truncated Notch 1 receptor construct (NΔECD), which does not require ligand binding for its activation and represents an immediate substrate of γ-secretase, and pCS2 Notch1 ICD-6MT plasmid encoding the intracellular domain of murine Notch1 (NICD) were gifts from Dr. R. Kopan (Washington University, St. Louis).

### GST protein cloning, purification, and LC-MS/MS analysis

The protocol used for GST protein cloning and purification was adapted from [53]. Briefly, DNA fragments encoding human presenilin 1 N-terminus (PS1 NT) (amino acids 1–80), PS1 loop 1–2 (amino acids 98–134), and PS1 loop 6–7 (amino acids 263–376) were sub-cloned into the glutathione S-transferase (GST) expression vector pGEX-6P-2 (GE Healthcare Life Sciences, Pittsburgh, PA) to create corresponding PS1 fragment-GST fusion peptides. For GST protein purification, *Escherichia coli* BL21 (*fhuA2 [lon] ompT gal [dcm] ΔhsdS*) cells were transformed with the plasmids and the protein expression was induced by the addition of 1 mM isopropyl-D-thiogalactoside (IPTG). The bacterial cultures were centrifuged at 3500 xg for 20 minutes at 4 °C, the pellet was resuspended in TBST and the bacterial suspensions were sonicated. The lysates were clarified by centrifugation at 14,000 xg for 15 minutes at 4 °C, and then incubated with washed glutathione-Sepharose 4B beads.

Mouse brain lysates prepared in 1 % Triton X-100 ice-cold lysis buffer (see below) were used for MS analysis. The soluble supernatant fraction was divided into equal aliquots that were incubated with GST-PS1 peptides, immobilized on a column in Ca^2+^-free or 2 mM CaCl_2_ conditions. The recombinant GST peptide was used as a control. The bound proteins were eluted with 2xSDS NuPage sample buffer (Life Technologies, Grand Island, NY) and separated by electrophoresis using 4–12 % 1.5 mm Bis-Tris NuPage gels (Life Technologies, Grand Island, NY). The gels were stained with coomassie blue stain (Life Technologies, Grand Island, NY), and several bands that displayed differential protein profile between GST-control and GST-PS1 pull-down, between GST-PS1 pull-down from brain lysates vs. lysis buffer, and between the Ca^2+^(+) and Ca^2+^(-) conditions were excised, and sent to Harvard University’s Taplin Biological Mass Spectrometry Facility (https://taplin.med.harvard.edu/) for analysis.

### Mouse brain lysates

Brain cortices were dissected from 2- to 3-month-old wild type (wt) CD1 mice (Charles River Laboratories, Wilmington, MA) and homogenized in a buffer containing 50 mM HEPES, 125 mM NaCl, 0.1 mM EDTA, 100 mM sucrose, protease inhibitors (Roche, Indianapolis, Indiana) and 1 % Triton X-100 (Tx-100) or 1 % 3-[(3-cholamidopropyl) dimethylammonio]-2-hydroxy-1-propanesulfonate (CHAPSO)). Brain lysates were incubated for 1 hour at 4 °C and centrifuged at 14,000xg to collect the soluble fractions, which were subjected to mass spectrometry (MS), immunoprecipitation (IP) and western blot analyses. All experiments that involve use of the mouse brain tissue were approved by the Subcommittee for Research Animal Care at the Massachusetts General Hospital.

### Synaptoneurosome (SNS) extraction

SNSs were isolated as described previously [54] with minor modifications. In brief, cortices from 3-month-old CD1 male mice were mechanically homogenized in 1.5 ml ice cold buffer A (25 mM HEPES, 120 mM NaCl, 5 mM KCl, 1 mM MgCl_2_) supplemented with 2 mM dithiothreitol (DTT), protease inhibitor (Roche, Indianapolis, Indiana), and phosphatase inhibitor cocktails 2 and 3 (Sigma-Aldrich, St. Louis, MO). Samples were filtered through two layers of 80 μm nylon filters (Millipore, Temecula, CA) to remove cellular debris. A total of 200 μl from each sample was saved, mixed with 200 μl water and 70 μl 10 % SDS, passed through a 27-gauge needle, and boiled for 5 minutes to be used as a total extract. The remaining homogenates were filtered through a 5 μm Supor membrane filter (Pall Corp., Port Washington, NY) to remove large organelles and nuclei, and the filtrates were centrifuged at 1000xg for 10 minutes to pellet SNSs. The pellets were resuspended in buffer A and used for immunostaining or were lysed in buffer B (50 mM Tris, 1.5 % SDS, and 2 mM DTT) and used for immunoprecipitation and western blotting.

### Antibodies

The following primary antibodies were used: anti-PS1 NT raised against the N-terminus of human PS1 (H-70, sc-7104, Santa Cruz Biotechnology, Santa Cruz, CA, and MAB1563, Millipore, Temecula, CA); anti-PS1 CT raised against the C-terminus of human PS1 (D3901, Cell Signaling Technology, Danvers, MA); anti-PS1 loop raised against the loop domain between transmembrane domains 6 and 7 of human PS1 (2094-1, Epitomics, Burlingame, CA, and MAB5232, Millipore, Temecula, CA); ASV30 anti-synaptotagmin 1 (Syt1) raised against rat brain synaptic junctional complexes (MA125568, Pierce, Rockford, IL); anti-Syt1 raised against the C-terminus of rat Syt1 (MAB5200, Millipore, Temecula, CA); anti-V5 (R960-25, Life Technologies, Grand Island, NY); anti-FLAG (F1804, Sigma-Aldrich, St. Louis, MO), anti-synapsin 1 (Syn1) (D12G5, Cell Signaling Technology, Danvers, MA); anti-nicastrin (PRS3985, Sigma-Aldrich, St. Louis, MO); anti-Pen-2 (P5622, Sigma-Aldrich, St. Louis, MO); anti-Aph1aL (PRB-550P, Covance, Dedham, MA); anti-amyloid precursor protein (APP) CT (A8717, Sigma-Aldrich, St. Louis, MO); anti-APP NT (MAB348, Millipore); anti-sAPPβ (18957, IBL America, Minneapolis, MN); anti-APP (597) (28055, IBL America, Minneapolis, MN); APP C66 (custom); anti-BACE1 (D10E5, Cell Signaling Technology, Danvers, MA); anti-BACE1 raised against the prodomain (LS-C51629, LifeSpan Biosciences, Seattle, WA); anti-actin (4968, Cell Signaling Technology, Danvers, MA), anti-Na^+^/K^+^ ATPase (AB7671, Abcam, Cambridge, MA); anti-Rab11 (610656, BD Biosciences); anti-Tgn46 (AB16059, Abcam, Cambridge, MA); anti-GM130 (610822, BD Biosciences, San Jose, CA); anti-calreticulin (612136, BD Biosciences, San Jose, CA), anti-GFP (632380, Clontech, Madison, WI), and anti-Myc (MA1-980, Pierce, Rockford, IL). Alexa Fluor 488 and Cy3-labeled corresponding secondary antibodies (Life Technologies, Grand Island, NY) were used for confocal microscopy imaging, and horseradish peroxidase (HRP)-conjugated ones (Pierce, Rockford, IL) for western blotting.

### Immunoprecipitation and western blotting

Hippocampal brain tissue from wt 3-month-old male CD1 mice was homogenized in 9 volumes of 1 % CHAPSO buffer (50 mM HEPES, 100 mM NaCl, pH 7.1 and 1 % CHAPSO) using plastic pestle homogenizers. Pelleted SNS fractions from wt mice brain cortex and cultured cells were homogenized by pipetting in 1 % CHAPSO or 1 % Tx-100 buffer (50 mM HEPES, 100 mM NaCl, pH 7.4, and 1 % Tx-100) and then passing through a 27-gauge needle. All lysis buffers were supplemented with HALT protease and phosphatase inhibitor cocktails (Fisher, Pittsburg, PA). The homogenates were rotated at 4 °C for 1 hour, centrifuged at 20,000xg for 20 minutes and the supernatants were collected as samples for IP. Total protein concentrations were determined by Thermo Scientific™ Pierce™ BCA™ Protein Assay (Pierce, Rockford, IL).

Aliquots of the supernatant containing equal amounts of total protein were incubated with anti-PS1 NT, anti-PS1 CT, anti-PS1 loop, or anti-Syt1 antibodies overnight at 4 °C with end-over-end rotation. Normal rabbit IgG or mouse anti-FLAG antibodies were used as negative controls. After the overnight incubation, 20–30 μl of Protein G Dynabeads (Life Technologies, Grand Island, NY) were added to each sample and incubated for 10 minutes at room temperature. The Dynabeads were collected and washed twice with 1 % CHAPSO buffer and once with wash buffer (50 mM HEPES, 100 mM NaCl, pH 7.4) or three times with 1 % Tx-100 buffer. Bound proteins were eluted by boiling at 95 °C for 5 minutes with 2xLDS sample buffer (Life Technologies, Grand Island, NY) containing 4 % β-mercaptoethanol.

Immunoprecipitated or total protein was separated by SDS-PAGE on 4–12 % or 10 % Bis-Tris NuPage polyacrylamide gels (Life Technologies, Grand Island, NY). After the electrophoresis, protein was transferred to nitrocellulose membranes (GE Healthcare Lifesciences, Pittsburgh, PA) and probed with respective primary and corresponding HRP-conjugated secondary antibodies. The blots were developed using ECL Western Blotting Substrate (Pierce, Rockford, IL) and exposed on X-ray films (Amersham Hyperfilm ECL). The intensity of respective protein bands was quantified using ImageJ 1.46c software. When necessary, antibodies were stripped using One Minute WB Advance Stripping Buffer (GM Biosciences, Rockville, MD).

### Cell cultures and transfection

Primary neuronal cultures were obtained from 16- to 18-day-old embryos of CD1 wild-type mice. Cortical and hippocampal neurons were extracted using a Papain Dissociation Kit (Worthington Biochemical Corporation, Lakewood, NJ) and plated onto tissue culture grade dishes and slides coated with poly-D-lysine. Cells were cultured for 12–28 days in vitro (DIV) in Neurobasal Media supplemented with 2 % B27 supplement, 1 % penicillin/streptomycin, 1 % Glutamax (Life Technologies, Grand Island, NY) in 37 °C, 5 % CO_2_ incubator.

Rat pheochromocytoma cell line (PC12) stably expressing short hairpin RNA (shRNA) specifically silencing the expression of Syt1, referred to as Syt1 KD PC12, and the parental PC12 cell line were kindly provided by Dr. Amy B. Harkins (St. Louis University School of Medicine) [55]. The cells were grown in RPMI Medium 1640 supplemented with 10 % heat-inactivated horse serum, 5 % fetal bovine serum (FBS), and 1 % penicillin/streptomycin (Pen/Strep) (Life Technologies, Grand Island, NY) in a 37 °C, 5 % CO_2_ incubator. For biochemical or microscopy (optical imaging) analyses, they were plated into 10 cm Petri dishes (Becton Dickinson Labware, Franklin Lakes, NJ) or 35 mm glass-bottom culture dishes (MatTek Corp, Ashland, MA), respectively. When needed, cells were transfected with selected expression plasmids using Lipofectamine LTX and Plus reagents (Life Technologies, Grand Island, NY).

PS double-knockout (PS-/-) mouse embryonic fibroblasts (MEF) and PS-/- stably transfected with wt hu PS1 were provided by Dr. B. De Strooper (Belgium). Chinese hamster ovary (CHO) cell lines expressing human wt APP and PS1 (PS70) were gifts from Dr. Selkoe (Boston, MA). CHO cells stably expressing human wt APP and Syt1-V5 were generated in Dr. Kovacs lab (Boston, MA). All the cell lines were cultured in Opti-MEM® I Reduced Serum medium supplemented with 5 % FBS (Life Technologies, Grand Island, NY), and respective selection antibiotics in a 37 °C, 5 % CO_2_ incubator. When necessary, the cells were transfected with other expression plasmids using Lipofectamine LTX (Life Technologies, Grand Island, NY).

### Spectral Förster resonance energy transfer (FRET)

Primary neurons (12–14 days in vitro [DIV 12–14]) were cultured in phenol red-free culture medium and transfected with G-PS1-R expression plasmid as a reporter of PS1 conformational changes, or G-PS1, R-PS1, and G-R fusion constructs as controls for determining spectral FRET settings using Metadetector on a Zeiss LCM510 microscope as described [25]. For the time-lapse recording, neurons were imaged twenty-four hours after the transfection with G-PS1-R. An argon laser at 488 nm was used to excite GFP, and emitted fluorescence was detected within 513 ± 10.57 nm (GFP) and 598 ± 10.57 nm (RFP) spectral bandwidth of the Metadetector [25]. The fluorescence signal was recorded every ~30 seconds using a Zeiss LCM510 microscope equipped with 37 °C, 5 % CO_2_ incubation chamber.

The G-PS1-R transfected neurons were imaged for 5 minutes to set up the baseline RFP/GFP ratio. Then, 100 μl of 50 mM KCl or H_2_O were added directly into the dish or 10 μl of 1 M L-glutamate (Glu) (Sigma-Aldrich, St. Louis, MO) were squirted directly onto imaged neuron, and changes in the fluorescence intensity were recorded for up to 60 (KCl) or 15 (Glu) minutes post-treatment. In the control experiment, to prevent Ca^2+^ influx into the Glu-treated neurons, culture medium was replaced with Hanks Balanced Salt Solution without Ca^2+^ and Mg^2+^ (Life Technologies, Grand Island, NY).

The acquired images were analyzed using LSM Image Browser. A ratio of 598 nm (RFP) to 513 nm (GFP) was used as readout of the FRET efficiency (%E_FRET_) (R/G ratio), which reflects relative proximity between the RFP and the GFP fluorophores. A ratiometric pseudo-color image was produced using ImageJ 1.46c software, by dividing the average pixel fluorescence intensity of an image in the 598 nm spectral window by that in the 513 nm window, after subtracting the background fluorescence. The Look-Up Table (LUT) was applied by mapping image values to color-scale (16 colors) resulting in the pseudo-color image.

### Calcium imaging

To measure changes in intracellular Ca^2+^ levels, primary neurons were pre-loaded with 5 μM Oregon Green 488 BAPTA-1 AM (Life Technologies, Grand Island, NY) and imaged before and after KCl or Glu treatment. The images were captured every 3.95 seconds using time-lapse settings, following an excitation with a 488 Argon laser. The average intensities of the Oregon Green 488 BAPTA-1 AM pre-loaded cells were measured using ImageJ 1.46c software. The fluorescence intensities of the cells recorded after the treatment were normalized to the pre-treatment ones, after background fluorescence subtraction.

### Immunocytochemistry

Primary neurons were fixed in 4 % PFA, permeabilized in 0.1 % Tx-100 with 1.5 % normal donkey serum (Jackson ImmunoResearch Labs, West Grove, PA), and incubated overnight at 4 °C with the following primary antibodies: goat anti-PS1 loop and mouse anti-Syt1 for the PS1-Syt1 proximity assay and rabbit anti-APP CT and mouse anti-PS1 loop for the PS1-APP interaction assay. Corresponding Alexa Fluor 488- or Cy3-labeled secondary antibodies were added for 1 hour at room temperature before mounting the coverslips with VectaShield mounting medium (Vector Laboratories, Inc., Burlingame, CA).

### Fluorescent lifetime imaging microscopy (FLIM)

The proximity between fluorophore labeled endogenous PS1 and Syt1, PS1 and APP, or PS1 NT and CT was evaluated by a previously validated FLIM assay [22, 56]. Briefly, pulsing Chameleon Ti:Sapphire laser (Coherent Inc., Santa Clara, CA) was used to excite Alexa Fluor 488 donor fluorophore (two-photon excitation at 780 nm wavelength). The baseline lifetime (*t*1) of the Alexa Fluor 488 fluorophore was measured in the absence of the Cy3 acceptor fluorophore (negative control, FRET absent). Donor fluorophore lifetimes were recorded using a high-speed photomultiplier tube (MCP R3809; Hamamatsu, Bridgewater, NJ) and a fast time-correlated single-photon counting acquisition board (SPC-830; Becker & Hickl, Berlin, Germany). In the presence of the acceptor fluorophore, excitation of the donor fluorophore yields reduced donor emission energy if the donor and the acceptor are less than 5–10 nm apart (FRET present). This results in a characteristic shortening of the donor fluorophore lifetime (*t*2). The acquired FLIM data were analyzed using SPC Image software (Becker & Hickl, Berlin, Germany). The %E_FRET_ was calculated using the following equation: %E_FRET_ = 100*(*t*1-*t*2)/*t*1 [57]. The degree of the donor fluorophore lifetime shortening (or increase in the %E_FRET_) correlates with the increase in the proximity between the PS1 NT and CT, or PS1 and either Syt1 or APP molecules.

### Immunoelectron microscopy

Adult CD1 mice were perfused with 4 % paraformaldehyde (PFA) before the cortices were excised and 1 mm-thick slices were fixed in a mixture of 0.1 % tetroxide, dehydrated, and embedded in araldite/dodecynyl succinic anhydride (DDSA) resin (Electron Microscopy Sciences, Hatfield, PA). Then, 70 nm-thick sections were cut on an ultracut microtome (Leica) and dried onto formar/carbon-coated mesh electron microscopy (EM) grids prior to immunostaining with anti-PS1 C-terminal fragment primary antibody (Sigma, P7854), followed by 6 nm-Ag-particles labeled secondary antibody (Aurion). Grids were negatively stained with 1 % filtered uranyl acetate in 70 % ethanol. The samples were examined and the images acquired using a JEOL 100S electron microscope (JEOL, USA, Peabody MA).

### ELISA for Aβ40 and Aβ42

To determine Aβ levels secreted into conditioned medium, culture medium of each sample was replaced by an equal amount of serum-free Advanced DMEM supplemented with 2 % GlutaMAX for PC12 cell lines and fresh Neurobasal growth medium supplemented with 2 % GlutaMAX and 1 % B27 for mouse primary neurons, respectively (Life Technologies, Grand Island, NY). Aβ40 and Aβ42 levels were measured using human/rat Aβ40 and Aβ42 enzyme-linked immunosorbent assay (ELISA) kits (Wako, Japan) according to the manufacturer’s instructions. Each value recorded in ELISA was normalized to the protein concentration of the corresponding cell extract measured with a BCA protein assay (Pierce, Rockford, IL).

### Cell-free γ-secretase assay

The crude homogenates of cellular membranes were prepared from parental or Syt1 KD PC12 cells in 20 mM HEPES (pH 7.4) containing protease inhibitor cocktail (Roche, Indianapolis, IN). The lysates were centrifuged at 3000xg for 15 minutes to remove cellular debris and nuclei. The supernatant was collected and centrifuged further at 100,000xg for 1 hour in a L8-80 M ultracentrifuge equipped with a Ti70.1 rotor (Beckman). Equal amounts of 1 % CHAPSO-solubilized membranes were incubated with C100-FLAG as a substrate (kindly provided by Dr. M.S. Wolfe, BWH, Boston, MA) for 4 hours at 37 °C. After the incubation, the samples were placed on ice to stop the reaction. For control purposes the reaction was carried out at 4 °C or in the presence of γ-secretase inhibitors.

### Fractionation of subcellular membrane vesicles by discontinuous OptiPrep density gradient

Preparation of total cellular membranes and subcellular fractionation were based on procedures described previously [58] with minor modifications. In brief, parental or Syt1 KD PC12 cells grown to confluence were washed and harvested in cold HEPES buffer (25 mM HEPES, 150 mM NaCl, 1 mM DTT, 2 mM EGTA, pH7.4). Cellular pellets were resuspended in the HEPES buffer and homogenized by passing through a 27-gauge needle. Total cellular membranes isolated from parental or Syt1 KD PC12 cells were loaded on a step OptiPrep gradient of 10–30 % with 2.5 % increments and centrifuged in SW 41 Ti rotor (Beckman Coulter Life Sciences, Indianapolis, IN) at 100,000xg at 4 °C for 16 hours. Then, 13 fractions were collected from the bottom of each centrifuge tube by puncture and equal amounts of the fractions were subjected to SDS-PAGE and western blotting.

### Cell surface protein isolation

Biotin-labeled cell surface proteins were isolated from parental and Syt1 KD PC12 cells using a Thermo Scientific™ Pierce™ Cell Surface Protein Isolation Kit (Pierce, Rockford, IL). Briefly, cells grown to confluence in collagen-coated dishes were incubated with sulfo-NHS-SS-Biotin, dissolved in cold PBS, for 30 minutes at 4 °C. After the incubation, the reaction was quenched by adding a quenching solution.

The cells were collected, washed twice with TBS, and lysed using a proprietary lysis reagent containing zwitterionic detergent in 25 nM bicine buffer (pH7.6). The biotin-labeled protein was isolated with NeutrAvidin agarose by incubating the cell lysates with SDS-PAGE sample buffer (Pierce, Rockford, IL) containing 50 mM DTT. All the reagents, unless stated otherwise, were provided with the kit. Protein concentration in each sample was determined using BCA Protein Assay (Pierce, Rockford, IL) and equal amounts of protein were subjected to SDS-PAGE and western blotting.

### Cycloheximide (CHX) pulse-chase assay

Relative stability of the protein was estimated using a CHX pulse-chase assay. Parental and Syt1 KD PC12 cells were treated with 20 μg/ml CHX (Sigma-Aldrich, St. Louis, MO), diluted in the growth medium. The cells were collected in 0, 2, 4, 8, 24, and 48 hours or 0, 15, 30, 45, and 90 minutes following the addition of CHX, and sonicated in 1 % Tx-100 buffer (50 mM HEPES, 100 mM NaCl, pH7.4). The extracted proteins were analyzed by SDS-PAGE and western blotting. The intensity of each band was quantified using ImageJ 1.46c software and normalized to the levels of a respective protein at 0 time point. The data were plotted on the graph, fitted with a one-phase decay curve, and analyzed with 2-way ANOVA followed by multiple comparison Bonferroni post-test, using GraphPad Prism 6 software.

### Notch1 processing assay

Parental and Syt1 KD PC12 cells were transfected with construct encoding Myc-tagged Notch1 with deleted extracellular domain (NΔECD) or Myc-tagged Notch1 intracellular domain (NICD) as a control. To control for the specificity of the γ-secretase activity assay, the cells were treated for 12 hours with 500 nM γ-secretase inhibitor, N-[N-(3,5-difluorophenacetyl)-L-alanyl]-S-phenylglycine t-butyl ester (DAPT), or DMSO added to the medium 12 hours after the transfection. Total protein was extracted from the cells, resolved by SDS-PAGE, and NΔECD and NICD were detected using anti-Myc antibody (Pierce, Rockford, IL). The intensities of the bands corresponding to NΔECD and NICD were quantified using ImageJ 1.46c software, and the NICD/NΔECD ratio was compared between parental and Syt1 KD cells.

### Statistics

To determine whether the R/G ratio in neurons treated with either KCl or glutamate differs from the one in cells treated with the vehicle control, a mixed model for the log ratio with repeated measures and random effects was constructed and tested. Fixed effects were treatment, pre- vs. post-treatment indicator, and the interaction between them. Repeated measures over time employed a covariance structure of first order auto-regressive moving average (ARMA(1,1)). To allow for different baseline levels for individual neurons, a random intercept was included in the model. To answer the question whether the increased R/G ratio in the cells treated with Glu actually goes back to the baseline, we fitted a piecewise mixed linear model for the treated group. This model includes linear components broken down by the time interval points 0 to 5 (before treatment), 5 to 6 (immediately after treatment) and 6 on as fixed effects and random effects to allow different slopes for individual cells and repeated measures with auto-regressive covariance structure. In order to test a decrease in the ratio back to pre-treatment levels, piecewise time components were designed to test a non-zero slope. The results from the model suggest a slope that is significantly different from 0 for the interval point 5 to 6 (*p*-value 0.0001). The parameter estimate for this slope is positive, which is in line with the hypothesized increase in the R/G ratio between the time of treatment initiation and the time point 6. The slope for the time interval point 6 on is also significantly different from 0 (*p*-value 0.024) with a negative parameter estimate, suggesting again the hypothesized decrease in R/G ratio. In addition, the initial time interval in the model, which corresponds with the baseline pre-treatment period, does not present with a slope significantly different than 0, suggesting no trend in the ratio, which is an expected outcome under no activity.

Statistical analysis of the FLIM data was performed using StatView for Windows, Version 5.0.1 (SAS Institute, Inc.). For quantitative western blotting, the intensities of the bands corresponding to target proteins were measured using ImageJ 1.46c software and compared between the samples. Statistics were calculated with Microsoft Office Excel 2007 or GraphPad® Prism 6 software using a two-tailed unpaired student-test, Mann-Whitney’s *U* test or 2-way ANOVA with Bonferroni multiple comparison post-test. *P*-value of < 0.05 was a predetermined threshold for statistical significance. The data were recorded from at least three different samples obtained on separate days; n indicates biological replicates for each experiment.

### Ethics approval

All experiments involving use of the mouse brain tissue were approved by the Subcommittee for Research Animal Care at the Massachusetts General Hospital.

### Availability of supporting data

The datasets supporting the conclusions of this article are available in the IMEx consortium - IntAct [X] repository [IM-25035] and included in the article as additional files. Additional file 7 presents individual values recorded in the experiments where n < 6. (XLSX 270 kb)

## Additional files

Additional file 1: Spectral FRET analysis of the GFP and RFP intensities. Primary neurons were transfected with GFP-PS1 (negative FRET control, *n* = 12 cells), RFP-GFP fusion (R-G, positive FRET control, *n* = 24) or GFP-PS1-RFP (PS1 conformation FRET probe, *n* = 10). The GFP was excited by argon laser at 488 nm wavelength, and the emission intensities of GFP and RFP within the 513 ± 10.57 nm and 598 ± 10.57 nm spectral bandwidth of the Metadector, respectively, were collected every 3 minutes for the duration of 33 minutes (middle and bottom graphs, respectively). The arrow indicates the time point of 50 mM KCl stimulation. No significant change in the GFP or RFP fluorescence emission intensity (no photobleaching), or the R/G ratio was observed in the G-PS1 and R-G transfected cells. The change in the R/G ratio after KCl stimulation was detected only in G-PS1-R transfected cells due to increased FRET efficiency/change in PS1 conformation. The black line shows mean ± SEM values. (TIF 1663 kb) Additional file 2: Proteins identified in mass spectrometry screen of mouse brain lysates for novel PS1 calcium-dependent interactors. Synaptotagmin 1 (Syt1) was detected in high calcium condition (calcium +) when GST-PS1 L6-7 and GST-PS1 NT recombinant peptides were used for pull-down. Excised band size and protein matches are indicated. (*n* = 3; Data from one representative experiment is shown). (XLSX 20 kb) Additional file 3: Knock-down and overexpression of Syt1 in PC12 cells. Representative western blot demonstrates successful stable RNAi-mediated knock-down of endogenous Syt1 and huSyt1-V5 overexpression in PC12 cell line. The graph shows quantitative analysis of the rat and rat + human Syt1 levels. Data are presented as mean ± SEM, *n* = 3 for Syt1 KD + Syt1-V5 and for parental PC12; and *n* = 4 for other conditions. Statistical significance was determined using unpaired student *t*-test; * *p* < 0.05, ** *p* < 0.01. (TIF 1863 kb) Additional file 4: PS1 and APP stability is not affected by Syt1 KD. A, Parental and Syt1 KD PC12 cells were treated with cycloheximide and harvested 0, 2, 4, 8, 24, and 48 hours after the treatment. Western blot presents degradation of PS1 over time. Anti-PS1 C-terminal antibody was used for detection. The quantitative analysis of PS1 levels reveals no difference in the half-life of PS1 in Syt1 KD PC12 cells compared to the parental control. Statistical significance was determined using 2-way ANOVA with Bonferroni post-test, *n* = 5. B, Parental and Syt1 KD PC12 cells were treated with cycloheximide and harvested 0, 15, 30, 45, and 90 minutes after the treatment. Western blot presents the level of APP over time. Anti-APP C-terminal antibody was used for detection. The quantitative analysis of APP levels revealed no difference in the half-life of APP in Syt1 KD PC12 cells compared to the parental control. Statistical significance was determined using 2-way ANOVA with Bonferroni post-test, *n* = 3. (TIF 3814 kb) Additional file 5: Overall transport fidelity is not altered by Syt1 KD. A, Western blots show distribution of Nct and Pen-2 in 13 subcellular fractions from parental and Syt1 KD cells. No differences in the subcellular distribution of Nct and Pen-2 were observed between parental and Syt1 KD cells. The enrichment of the respective subcellular compartments was determined by western blotting with anti-calreticulin, anti-GM130, anti-Tgn46, and anti-Rab11 antibodies. The fractions that correspond to the respective intracellular compartments are indicated above the western blots. B, Western blots show distribution of organelle (GM130, Tgn46, calreticulin, and Rab11) and synaptic vesicle marker, synaptophysin, in 13 subcellular fractions from parental and Syt1 KD cells. No significant differences in the subcellular distribution of organelles and synaptophysin (Syp) between parental and Syt1 KD cells were observed. (TIF 3749 kb) Additional file 6: Syt1 is not a substrate for γ-secretase. A, B, Western blots show levels of mature, immature, nascent Syt1, and Syt1 C-terminal fragments (Syt1 CTF) in mouse primary neurons (A) and PS-/- mouse embryonic fibroblasts (MEF) transfected with huSyt1-V5 and PS1 (as indicated) (B). Inhibition of the γ-secretase activity with DAPT and L685,458 did not alter Syt1 processing. GFP (transfection efficiency) and β actin (loading) are shown as controls. (TIF 1352 kb) Additional file 7: Supporting data values. The file presents individual values recorded in the experiments where *n* < 6. (XLSX 270 kb)

## Abbreviations

AD: Alzheimer’s disease
AICD: Amyloid intracellular domain
APP: Amyloid precursor protein
Aβ: Amyloid β
BACE1: β-secretase
CHO: Chinese hamster ovary cell line
CHX: Cycloheximide – inhibitor of protein biosynthesis
CT: C-terminus
CTF: C-terminal fragment
DAPT: N-[N-(3,5-Difluorophenacetyl)-L-alanyl]-S-phenylglycine t-butyl ester – γ-secretase inhibitor
DIV: Days in vitro
fAD: Familial Alzheimer’s disease
FL: Full length
FLIM: Fluorescence lifetime imaging microscopy
FRET: Förster Resonance Energy Transfer
G-PS1-R: GFP-PS1-RFP - PS1 tagged with green fluorescent protein at the N-terminus and RFP within the loop domain
GST: Glutathione S-transferase
KD: Knock-down
MEF: Mouse embryonic fibroblasts
MS: Mass spectrometry
NICD: Notch 1 intracellular domain
NT: N-terminus
NTF: N-terminal fragment
NΔECD: Notch1 with deleted extracellular domain
PC12: Rat pheochromocytoma cell line
PS1: Presenilin 1
PS1 L6-7: Loop domain between 6th and 7th transmembrane helices of PS1
R-G: Fusion of red and green fluorescent proteins
sAPPα/β: Secreted amyloid precursor protein α/β
SNS: Synaptoneurosomes
SV: Synaptic vesicles
Syt1: Synaptotagmin 1
wt: Wild type

## References

[1] De Strooper B, Saftig P, Craessaerts K, Vanderstichele H, Guhde G, Annaert W (1998). Deficiency of presenilin-1 inhibits the normal cleavage of amyloid precursor protein. Nature.

[2] Wolfe MS, Xia W, Ostaszewski BL, Diehl TS, Kimberly WT, Selkoe DJ (1999). Two transmembrane aspartates in presenilin-1 required for presenilin endoproteolysis and gamma-secretase activity. Nature.

[3] Vassar R, Bennett BD, Babu-Khan S, Kahn S, Mendiaz EA, Denis P (1999). Beta-secretase cleavage of Alzheimer’s amyloid precursor protein by the transmembrane aspartic protease BACE. Science.

[4] Querfurth HW, LaFerla FM (2010). Alzheimer’s disease. N Engl J Med.

[5] Mucke L, Selkoe DJ (2012). Neurotoxicity of amyloid beta-protein: synaptic and network dysfunction. Cold Spring Harb Perspect Med.

[6] Jack CR, Lowe VJ, Weigand SD, Wiste HJ, Senjem ML, Knopman DS (2009). Serial PIB and MRI in normal, mild cognitive impairment and Alzheimer’s disease: implications for sequence of pathological events in Alzheimer’s disease. Brain.

[7] Terry RD, Masliah E, Salmon DP, Butters N, DeTeresa R, Hill R (1991). Physical basis of cognitive alterations in Alzheimer’s disease: synapse loss is the major correlate of cognitive impairment. Ann Neurol.

[8] Knobloch M, Mansuy IM (2008). Dendritic spine loss and synaptic alterations in Alzheimer’s disease. Mol Neurobiol.

[9] Marcello E, Epis R, Di Luca M (2008). Amyloid flirting with synaptic failure: towards a comprehensive view of Alzheimer’s disease pathogenesis. Eur J Pharmacol.

[10] Kikuchi M, Hirosawa T, Yokokura M, Yagi S, Mori N, Yoshikawa E (2011). Effects of brain amyloid deposition and reduced glucose metabolism on the default mode of brain function in normal aging. J Neurosci.

[11] Kamenetz F, Tomita T, Hsieh H, Seabrook G, Borchelt D, Iwatsubo T (2003). APP processing and synaptic function. Neuron.

[12] Bero AW, Yan P, Roh JH, Cirrito JR, Stewart FR, Raichle ME (2011). Neuronal activity regulates the regional vulnerability to amyloid-beta deposition. Nat Neurosci.

[13] Li X, Uemura K, Hashimoto T, Nasser-Ghodsi N, Arimon M, Lill CM (2013). Neuronal activity and secreted amyloid beta lead to altered amyloid beta precursor protein and presenilin 1 interactions. Neurobiol Dis.

[14] Cirrito JR, Yamada KA, Finn MB, Sloviter RS, Bales KR, May PC (2005). Synaptic activity regulates interstitial fluid amyloid-beta levels in vivo. Neuron.

[15] Brose N, Petrenko AG, Sudhof TC, Jahn R (1992). Synaptotagmin: a calcium sensor on the synaptic vesicle surface. Science.

[16] Xu J, Pang ZP, Shin OH, Sudhof TC (2009). Synaptotagmin-1 functions as a Ca2+ sensor for spontaneous release. Nat Neurosci.

[17] Stevens CF, Sullivan JM (2003). The synaptotagmin C2A domain is part of the calcium sensor controlling fast synaptic transmission. Neuron.

[18] Sudhof TC (2012). Calcium control of neurotransmitter release. Cold Spring Harb Perspect Biol.

[19] Querfurth HW, Selkoe DJ (1994). Calcium ionophore increases amyloid beta peptide production by cultured cells. Biochemistry.

[20] Pierrot N, Ghisdal P, Caumont AS, Octave JN (2004). Intraneuronal amyloid-beta1-42 production triggered by sustained increase of cytosolic calcium concentration induces neuronal death. J Neurochem.

[21] Kim SH, Fraser PE, Westaway D, St George-Hyslop PH, Ehrlich ME, Gandy S (2010). Group II metabotropic glutamate receptor stimulation triggers production and release of Alzheimer’s amyloid(beta)42 from isolated intact nerve terminals. J Neurosci.

[22] Lleo A, Berezovska O, Herl L, Raju S, Deng A, Bacskai BJ (2004). Nonsteroidal anti-inflammatory drugs lower Abeta(42) and change presenilin 1 conformation. Nat Med.

[23] Berezovska O, Lleo A, Herl LD, Frosch MP, Stern EA, Bacskai BJ (2005). Familial Alzheimer’s disease presenilin 1 mutations cause alterations in the conformation of presenilin and interactions with amyloid precursor protein. J Neurosci.

[24] Isoo N, Sato C, Miyashita H, Shinohara M, Takasugi N, Morohashi Y (2007). Abeta42 overproduction associated with structural changes in the catalytic pore of gamma-secretase: common effects of pen-2 N-terminal elongation and fenofibrate. J Biol Chem.

[25] Uemura K, Lill CM, Li X, Peters JA, Ivanov A, Fan Z (2009). Allosteric modulation of PS1/gamma-secretase conformation correlates with amyloid beta(42/40) ratio. PLoS One.

[26] Fraering PC, LaVoie MJ, Ye W, Ostaszewski BL, Kimberly WT, Selkoe DJ (2004). Detergent-dependent dissociation of active gamma-secretase reveals an interaction between Pen-2 and PS1-NTF and offers a model for subunit organization within the complex. Biochemistry.

[27] Shao X, Davletov BA, Sutton RB, Sudhof TC, Rizo J (1996). Bipartite Ca2 + -binding motif in C2 domains of synaptotagmin and protein kinase C. Science.

[28] Fukumori A, Fluhrer R, Steiner H, Haass C (2010). Three-amino acid spacing of presenilin endoproteolysis suggests a general stepwise cleavage of gamma-secretase-mediated intramembrane proteolysis. J Neurosci.

[29] Qi-Takahara Y, Morishima-Kawashima M, Tanimura Y, Dolios G, Hirotani N, Horikoshi Y (2005). Longer forms of amyloid beta protein: implications for the mechanism of intramembrane cleavage by gamma-secretase. J Neurosci.

[30] Zhang C, Wu B, Beglopoulos V, Wines-Samuelson M, Zhang D, Dragatsis I (2009). Presenilins are essential for regulating neurotransmitter release. Nature.

[31] Cirrito JR, Kang JE, Lee J, Stewart FR, Verges DK, Silverio LM (2008). Endocytosis is required for synaptic activity-dependent release of amyloid-beta in vivo. Neuron.

[32] Takahashi RH, Milner TA, Li F, Nam EE, Edgar MA, Yamaguchi H (2002). Intraneuronal Alzheimer abeta42 accumulates in multivesicular bodies and is associated with synaptic pathology. Am J Pathol.

[33] Koffie RM, Meyer-Luehmann M, Hashimoto T, Adams KW, Mielke ML, Garcia-Alloza M (2009). Oligomeric amyloid beta associates with postsynaptic densities and correlates with excitatory synapse loss near senile plaques. Proc Natl Acad Sci U S A.

[34] Kuperstein I, Broersen K, Benilova I, Rozenski J, Jonckheere W, Debulpaep M (2010). Neurotoxicity of Alzheimer’s disease Abeta peptides is induced by small changes in the Abeta42 to Abeta40 ratio. EMBO J.

[35] Borchelt DR, Thinakaran G, Eckman CB, Lee MK, Davenport F, Ratovitsky T (1996). Familial Alzheimer’s disease-linked presenilin 1 variants elevate Abeta1-42/1-40 ratio in vitro and in vivo. Neuron.

[36] Scheuner D, Eckman C, Jensen M, Song X, Citron M, Suzuki N (1996). Secreted amyloid beta-protein similar to that in the senile plaques of Alzheimer’s disease is increased in vivo by the presenilin 1 and 2 and APP mutations linked to familial Alzheimer’s disease. Nat Med.

[37] De Strooper B (2007). Loss-of-function presenilin mutations in Alzheimer disease. Talking point on the role of presenilin mutations in Alzheimer disease. EMBO Rep.

[38] Serneels L, Van Biervliet J, Craessaerts K, Dejaegere T, Horre K, Van Houtvin T (2009). gamma-Secretase heterogeneity in the Aph1 subunit: relevance for Alzheimer’s disease. Science.

[39] Wahlster L, Arimon M, Nasser-Ghodsi N, Post KL, Serrano-Pozo A, Uemura K (2013). Presenilin-1 adopts pathogenic conformation in normal aging and in sporadic Alzheimer’s disease. Acta Neuropathol.

[40] Dolev I, Fogel H, Milshtein H, Berdichevsky Y, Lipstein N, Brose N (2013). Spike bursts increase amyloid-beta 40/42 ratio by inducing a presenilin-1 conformational change. Nat Neurosci.

[41] Gautam V, D’Avanzo C, Berezovska O, Tanzi RE, Kovacs DM (2015). Synaptotagmins interact with APP and promote Abeta generation. Mol Neurodegener.

[42] Chavez-Gutierrez L, Bammens L, Benilova I, Vandersteen A, Benurwar M, Borgers M (2012). The mechanism of γ-Secretase dysfunction in familial Alzheimer disease. EMBO J.

[43] Frykman S, Hur JY, Franberg J, Aoki M, Winblad B, Nahalkova J (2010). Synaptic and endosomal localization of active gamma-secretase in rat brain. PLoS One.

[44] Groemer TW, Thiel CS, Holt M, Riedel D, Hua Y, Huve J (2011). Amyloid precursor protein is trafficked and secreted via synaptic vesicles. PLoS One.

[45] Kandalepas PC, Sadleir KR, Eimer WA, Zhao J, Nicholson DA, Vassar R (2013). The Alzheimer’s beta-secretase BACE1 localizes to normal presynaptic terminals and to dystrophic presynaptic terminals surrounding amyloid plaques. Acta Neuropathol.

[46] Del Prete D, Lombino F, Liu X, D’Adamio L (2014). APP is cleaved by Bace1 in pre-synaptic vesicles and establishes a pre-synaptic interactome, via its intracellular domain, with molecular complexes that regulate pre-synaptic vesicles functions. PLoS One.

[47] Das U, Wang L, Ganguly A, Saikia JM, Wagner SL, Koo EH (2016). Visualizing APP and BACE-1 approximation in neurons yields insight into the amyloidogenic pathway. Nat Neurosci.

[48] Kohli BM, Pflieger D, Mueller LN, Carbonetti G, Aebersold R, Nitsch RM (2012). Interactome of the amyloid precursor protein APP in brain reveals a protein network involved in synaptic vesicle turnover and a close association with Synaptotagmin-1. J Proteome Res.

[49] Sannerud R, Declerck I, Peric A, Raemaekers T, Menendez G, Zhou L (2011). ADP ribosylation factor 6 (ARF6) controls amyloid precursor protein (APP) processing by mediating the endosomal sorting of BACE1. Proc Natl Acad Sci U S A.

[50] Burgos PV, Mardones GA, Rojas AL, da Silva LL, Prabhu Y, Hurley JH (2010). Sorting of the Alzheimer’s disease amyloid precursor protein mediated by the AP-4 complex. Dev Cell.

[51] Perez RG, Soriano S, Hayes JD, Ostaszewski B, Xia W, Selkoe DJ (1999). Mutagenesis identifies new signals for beta-amyloid precursor protein endocytosis, turnover, and the generation of secreted fragments, including Abeta42. J Biol Chem.

[52] Kuzuya A, Uemura K, Kitagawa N, Aoyagi N, Kihara T, Ninomiya H (2007). Presenilin 1 is involved in the maturation of beta-site amyloid precursor protein-cleaving enzyme 1 (BACE1). J Neurosci Res.

[53] Einarson MB, Pugacheva EN, Orlinick JR. Preparation of GST fusion proteins. CSH Protoc. 2007;2007:Pdb.prot4738. 10.1101/pdb.prot4738 21357069

[54] Hollingsworth EB, McNeal ET, Burton JL, Williams RJ, Daly JW, Creveling CR (1985). Biochemical characterization of a filtered synaptoneurosome preparation from guinea pig cerebral cortex: cyclic adenosine 3’:5’-monophosphate-generating systems, receptors, and enzymes. J Neurosci.

[55] Moore JM, Papke JB, Cahill AL, Harkins AB (2006). Stable gene silencing of synaptotagmin I in rat PC12 cells inhibits Ca2+ -evoked release of catecholamine. Am J Physiol Cell Physiol.

[56] Berezovska O, Bacskai BJ, Hyman BT (2003). Monitoring proteins in intact cells. Sci Aging Knowledge Environ.

[57] Lakowicz JR, Szmacinski H, Nowaczyk K, Berndt KW, Johnson M (1992). Fluorescence lifetime imaging. Anal Biochem.

[58] Li X, Donowitz M (2008). Fractionation of subcellular membrane vesicles of epithelial and nonepithelial cells by OptiPrep density gradient ultracentrifugation. Methods Mol Biol.

