# Supplementary figures and images for: Identification of the novel activity-driven interaction between synaptotagmin 1 and presenilin 1 links calcium, synapse, and amyloid beta
Source: BMC Biol. 2016 Mar 31;14:25. doi: 10.1186/s12915-016-0248-3 (PMC4818459; doi:10.1186/s12915-016-0248-3)

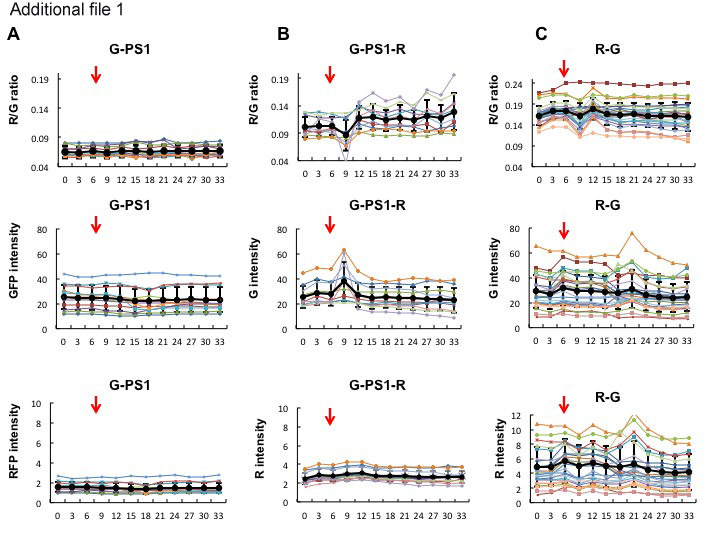

Supplement: Additional file 1: — Spectral FRET analysis of the GFP and RFP intensities. Primary neurons were transfected with GFP-PS1 (negative FRET control, n = 12 cells), RFP-GFP fusion (R-G, positive FRET control, n = 24) or GFP-PS1-RFP (PS1 conformation FRET probe, n = 10). The GFP was excited by argon laser at 488 nm wavelength, and the emission intensities of GFP and RFP within the 513 ± 10.57 nm and 598 ± 10.57 nm spectral bandwidth of the Metadector, respectively, were collected every 3 minutes for the duration of 33 minutes (middle and bottom graphs, respectively). The arrow indicates the time point of 50 mM KCl stimulation. No significant change in the GFP or RFP fluorescence emission intensity (no photobleaching), or the R/G ratio was observed in the G-PS1 and R-G transfected cells. The change in the R/G ratio after KCl stimulation was detected only in G-PS1-R transfected cells due to increased FRET efficiency/change in PS1 conformation. The black line shows mean ± SEM values. (TIF 1663 kb) [file 12915_2016_248_MOESM1_ESM.tif]

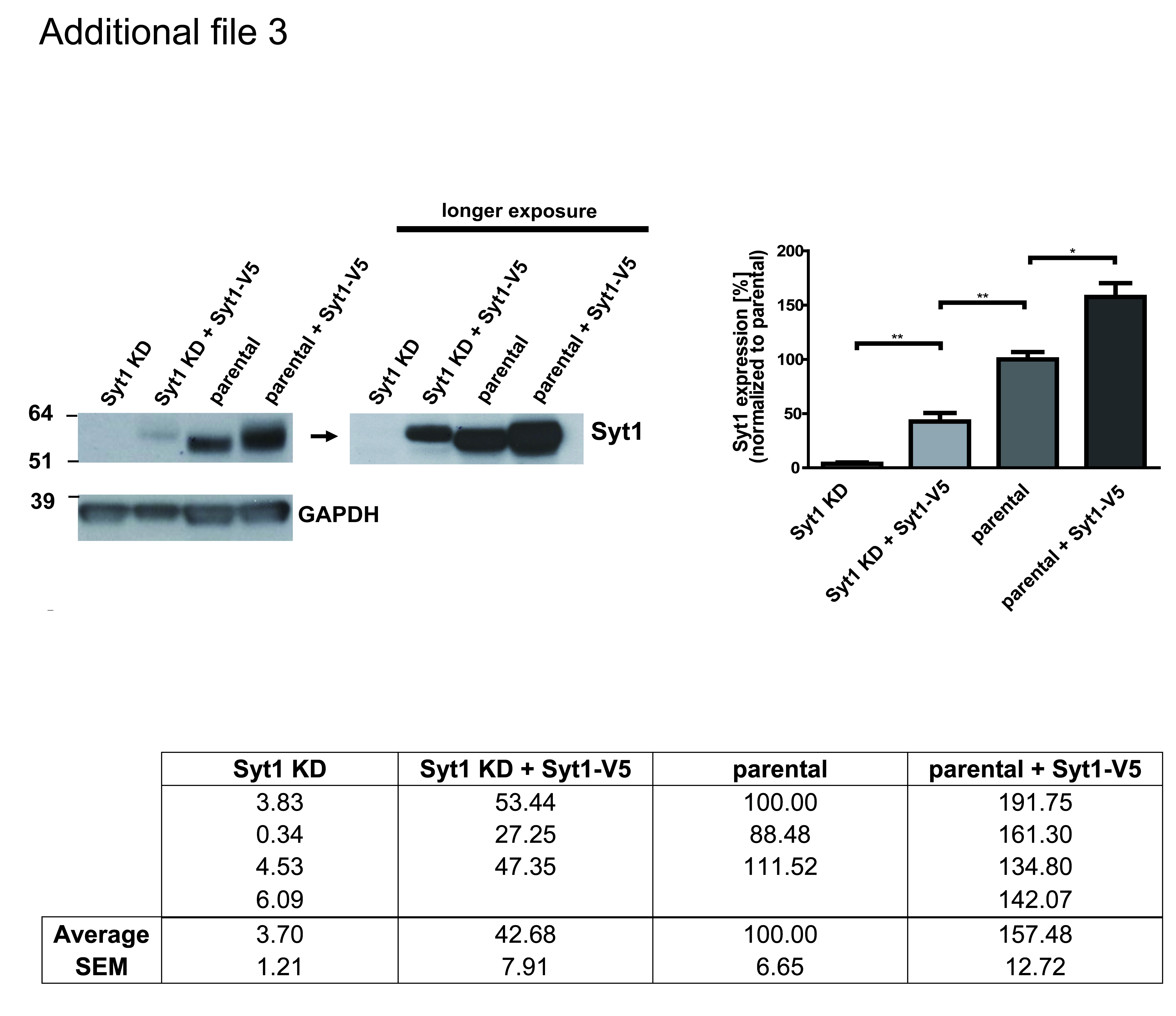

Supplement: Additional file 3: — Knock-down and overexpression of Syt1 in PC12 cells. Representative western blot demonstrates successful stable RNAi-mediated knock-down of endogenous Syt1 and huSyt1-V5 overexpression in PC12 cell line. The graph shows quantitative analysis of the rat and rat + human Syt1 levels. Data are presented as mean ± SEM, n = 3 for Syt1 KD + Syt1-V5 and for parental PC12; and n = 4 for other conditions. Statistical significance was determined using unpaired student t-test; * p < 0.05, ** p < 0.01. (TIF 1863 kb) [file 12915_2016_248_MOESM3_ESM.tif]

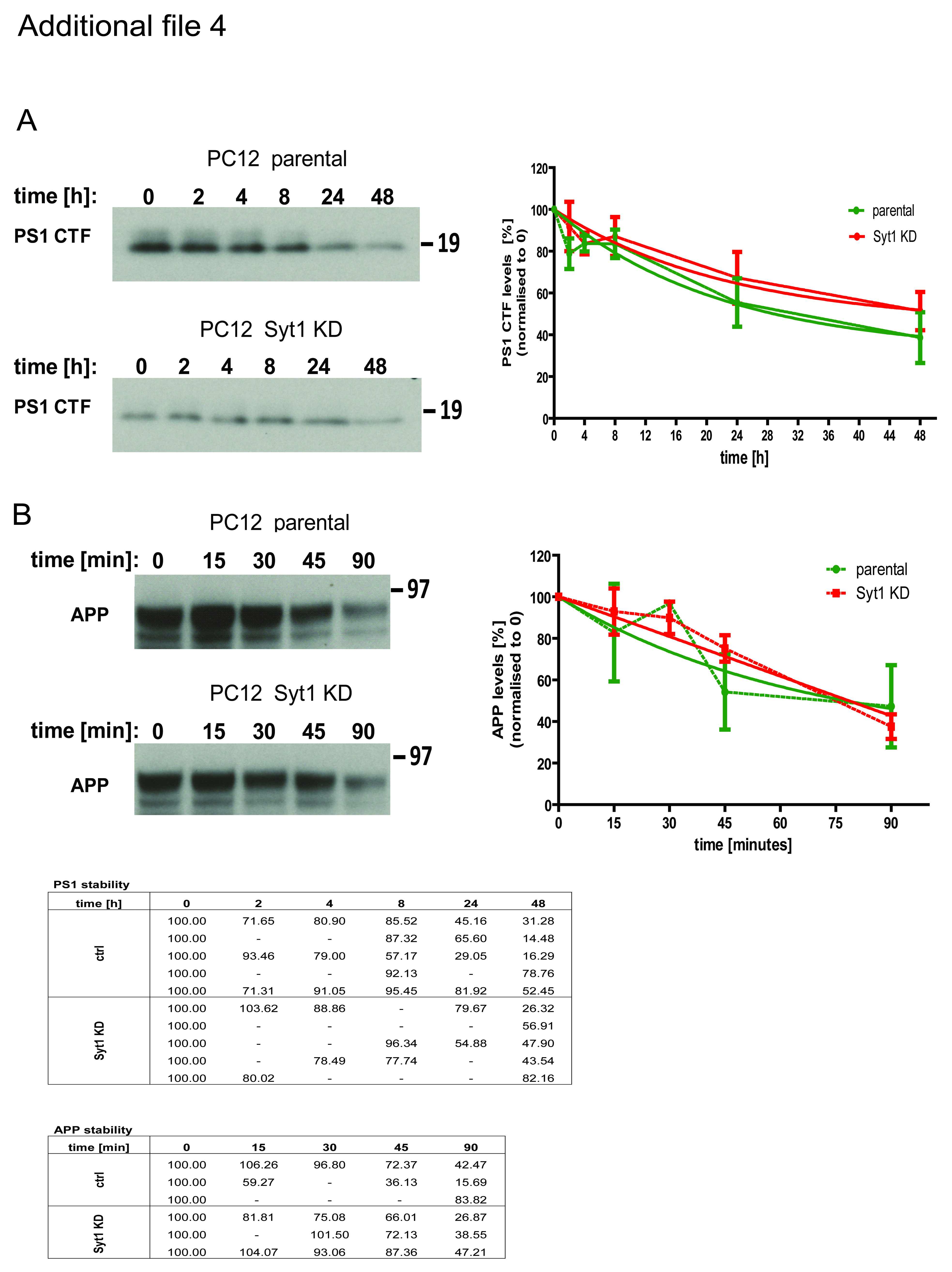

Supplement: Additional file 4: — PS1 and APP stability is not affected by Syt1 KD. A, Parental and Syt1 KD PC12 cells were treated with cycloheximide and harvested 0, 2, 4, 8, 24, and 48 hours after the treatment. Western blot presents degradation of PS1 over time. Anti-PS1 C-terminal antibody was used for detection. The quantitative analysis of PS1 levels reveals no difference in the half-life of PS1 in Syt1 KD PC12 cells compared to the parental control. Statistical significance was determined using 2-way ANOVA with Bonferroni post-test, n = 5. B, Parental and Syt1 KD PC12 cells were treated with cycloheximide and harvested 0, 15, 30, 45, and 90 minutes after the treatment. Western blot presents the level of APP over time. Anti-APP C-terminal antibody was used for detection. The quantitative analysis of APP levels revealed no difference in the half-life of APP in Syt1 KD PC12 cells compared to the parental control. Statistical significance was determined using 2-way ANOVA with Bonferroni post-test, n = 3. (TIF 3814 kb) [file 12915_2016_248_MOESM4_ESM.tif]

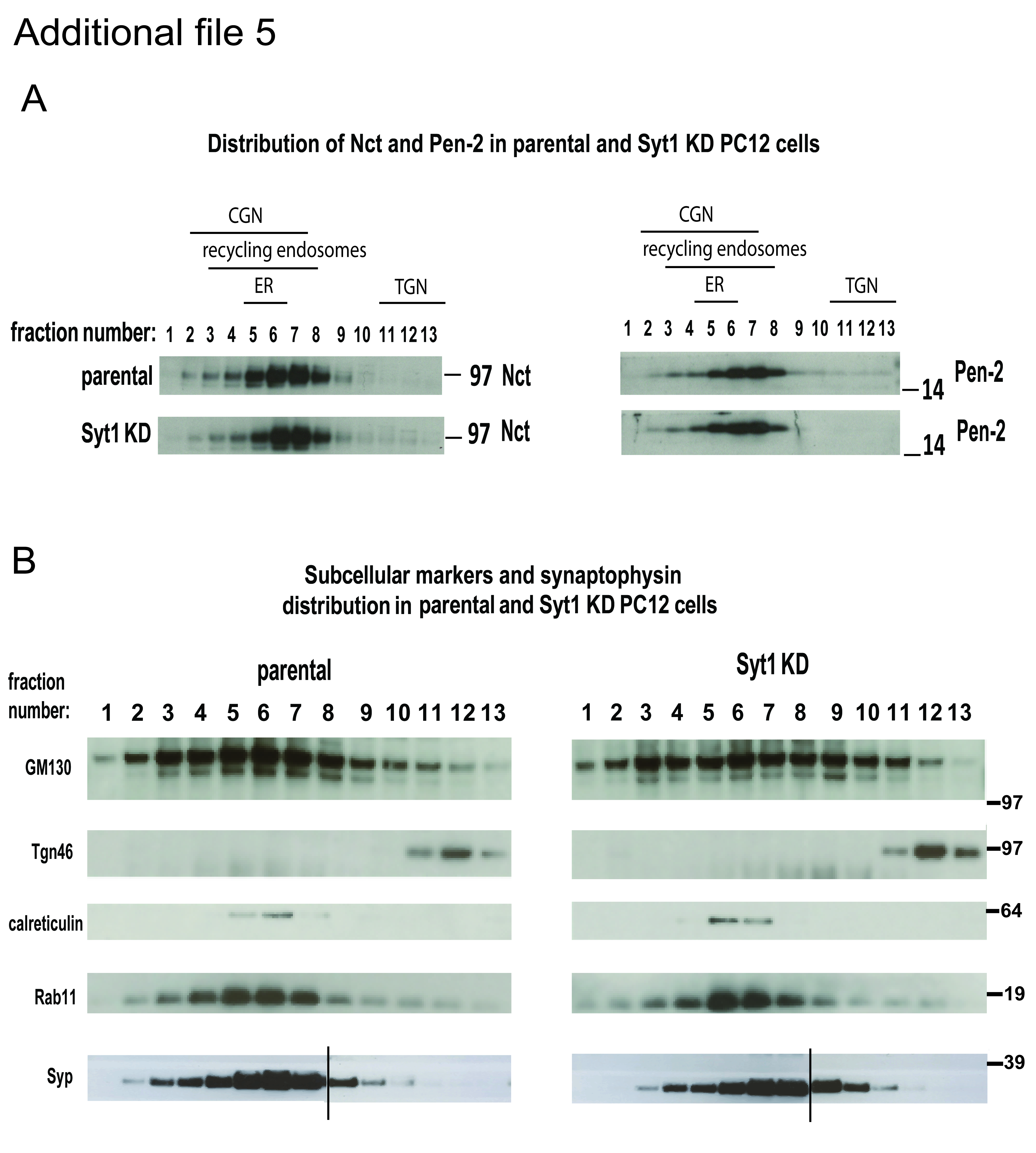

Supplement: Additional file 5: — Overall transport fidelity is not altered by Syt1 KD. A, Western blots show distribution of Nct and Pen-2 in 13 subcellular fractions from parental and Syt1 KD cells. No differences in the subcellular distribution of Nct and Pen-2 were observed between parental and Syt1 KD cells. The enrichment of the respective subcellular compartments was determined by western blotting with anti-calreticulin, anti-GM130, anti-Tgn46, and anti-Rab11 antibodies. The fractions that correspond to the respective intracellular compartments are indicated above the western blots. B, Western blots show distribution of organelle (GM130, Tgn46, calreticulin, and Rab11) and synaptic vesicle marker, synaptophysin, in 13 subcellular fractions from parental and Syt1 KD cells. No significant differences in the subcellular distribution of organelles and synaptophysin (Syp) between parental and Syt1 KD cells were observed. (TIF 3749 kb) [file 12915_2016_248_MOESM5_ESM.tif]

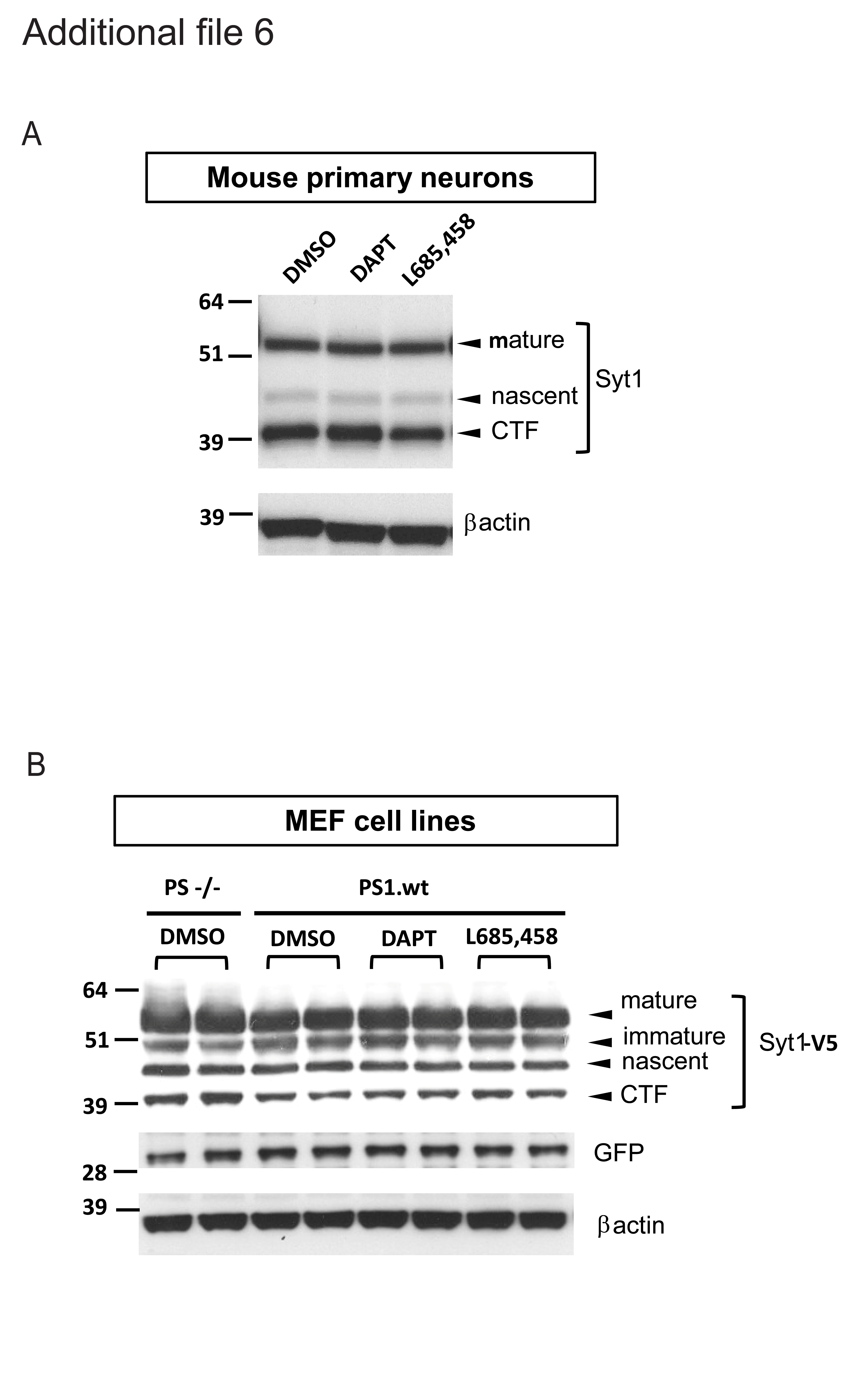

Supplement: Additional file 6: — Syt1 is not a substrate for γ-secretase. A, B, Western blots show levels of mature, immature, nascent Syt1, and Syt1 C-terminal fragments (Syt1 CTF) in mouse primary neurons (A) and PS-/- mouse embryonic fibroblasts (MEF) transfected with huSyt1-V5 and PS1 (as indicated) (B). Inhibition of the γ-secretase activity with DAPT and L685,458 did not alter Syt1 processing. GFP (transfection efficiency) and β actin (loading) are shown as controls. (TIF 1352 kb) [file 12915_2016_248_MOESM6_ESM.tif]
